# Supplementary material for: The role of information communication technologies as a moderator of knowledge creation and knowledge sharing in improving the quality of healthcare services
Source: PLoS One. 2022 Aug 3;17(8):e0272346. doi: 10.1371/journal.pone.0272346 (PMC9348677; doi:10.1371/journal.pone.0272346)
Supplement: Attachment — Submitted filename: PONE-D-21-25036_reviewer.pdf [file pone.0272346.s002.pdf]

PLOS ONE

The role of information communication technologies as a moderator of knowledge creation and knowledge transfer in improving the quality of healthcare services

--Manuscript Draft--

**Manuscript Number:**	PONE-D-21-25036
**Article Type:**	Research Article
**Full Title:**	The role of information communication technologies as a moderator of knowledge creation and knowledge transfer in improving the quality of healthcare services
**Short Title:**	ICT as a moderator of knowledge creation and transfer for improved healthcare quality
**Corresponding Author:**	Ivan Radević, Ph.D.<br>University of Montenegro, Faculty of Economics Podgorica<br>Podgorica, MONTENEGRO
**Keywords:**	quality of healthcare services; knowledge management; information and communication technologies; knowledge creation; knowledge transfer
**Abstract:**	This study examines the role of knowledge creation, knowledge transfer and information communication technologies, which are organizational factors that influence the quality of healthcare services. In today's knowledge-intensive environment, understanding and gaining in-depth knowledge on how to improve the quality of healthcare services is gaining in importance and recognition. Quantitative data collected in 2019 with 151 respondents employed in healthcare organizations was used. Running a series of hierarchical linear regression models, we found a significant positive relationship between knowledge creation and quality of healthcare services, and a significant positive relationship between knowledge transfer and quality of healthcare services. Empirical data additionally provides support for information communication technologies that act as a moderator both in the relationship between knowledge creation and knowledge transfer with quality of healthcare services. With our data, we provide empirical backing for the impact of knowledge creation, knowledge transfer and information communication technologies on the quality of healthcare services that are provided by Montenegrin healthcare organizations. Our paper offers theoretical and practical implications derived from our research study.
**Order of Authors:**	Simon Colnar
	Ivan Radević, Ph.D.
	Nikola Martinović
	Anđelko Lojpur
	Vlado Dimovski
**Additional Information:**	
**Question**	**Response**
**Financial Disclosure**<br><br>Enter a financial disclosure statement that describes the sources of funding for the work included in this submission. Review the submission guidelines for detailed requirements. View published research articles from *PLOS ONE* for specific examples.<br><br>This statement is required for submission and **will appear in the published article** if the submission is accepted. Please make	This work was supported by the Slovenian Research Agency, Program P5-0364 – The Impact of Corporate Governance, Organizational Learning, and Knowledge Management on Modern Organization.<br><br>The funders had no role in study design, data collection and analysis, decision to publish, or preparation of the manuscript.

sure it is accurate.<br><br>**Unfunded studies**<br>Enter: *The author(s) received no specific funding for this work.*<br><br>**Funded studies**<br>Enter a statement with the following details:<br>• Initials of the authors who received each award<br>• Grant numbers awarded to each author<br>• The full name of each funder<br>• URL of each funder website<br>• Did the sponsors or funders play any role in the study design, data collection and analysis, decision to publish, or preparation of the manuscript?<br>• **NO** - Include this sentence at the end of your statement: *The funders had no role in study design, data collection and analysis, decision to publish, or preparation of the manuscript.*<br>• **YES** - Specify the role(s) played.<br><br>* typeset	
**Competing Interests**<br><br>Use the instructions below to enter a competing interest statement for this submission. On behalf of all authors, disclose any competing interests that could be perceived to bias this work—acknowledging all financial support and any other relevant financial or non-financial competing interests.<br><br>This statement is required for submission and **will appear in the published article** if the submission is accepted. Please make sure it is accurate and that any funding sources listed in your Funding Information later in the submission form are also declared in your Financial Disclosure statement.<br><br>View published research articles from *PLOS ONE* for specific examples.	The authors have declared that no competing interests exist.

Powered by Editorial Manager® and ProduXion Manager® from Aries Systems Corporation

**NO authors have competing interests**<br><br>Enter: *The authors have declared that no competing interests exist.*<br><br>**Authors with competing interests**<br><br>Enter competing interest details beginning with this statement:<br><br>*I have read the journal's policy and the authors of this manuscript have the following competing interests: [insert competing interests here]*<br><br>* typeset	
**Ethics Statement**<br><br>Enter an ethics statement for this submission. This statement is required if the study involved:<br><br>• Human participants<br>• Human specimens or tissue<br>• Vertebrate animals or cephalopods<br>• Vertebrate embryos or tissues<br>• Field research<br><br>Write "N/A" if the submission does not require an ethics statement.<br><br>General guidance is provided below. Consult the submission guidelines for detailed instructions. **Make sure that all information entered here is included in the Methods section of the manuscript.**	University of Crna Gora/Montenegro (UCG) Committee for ethical issues (Etički odbor) deals exclusively with the issues of disciplinary measures, related to ethical breaches in the areas of teaching, student evaluation, plagiarism in research and cases of potential corruptive behavior at UCG. At UCG - Faculty of Economics, ethical approval of research is obtained either from the dean, or one of vice-deans, in an informal procedure. The practice of faculty administration in this matter includes the review of the objectives of the empirical research, the nature of data collection and the private information collected (if any), leading to the decision to approve the planned survey and the research design. We can supply a letter from the dean, or the vice-dean, if required. Data collection has been done via physical interviews (in 2019, before the Covid-19 pandemic). In our research, participation was entirely voluntary and anonymous. Our survey(s) did not include any opportunities to identify the individual responses and link it to the respondents' identities. Answers to demographic data, which were asked from the participants, were optional. A written introduction, uploaded on a tablet device, has been shown to the participants, prior to the beginning of interviews. It consisted of statements, related to the data collection policy, management of demographic data and the guarantee of anonymity and the cumulative (statistical) reporting of research results.

Powered by Editorial Manager® and ProduXion Manager® from Aries Systems Corporation

**Format for specific study types**<br><br>**Human Subject Research (involving human participants and/or tissue)**<br>• Give the name of the institutional review board or ethics committee that approved the study<br>• Include the approval number and/or a statement indicating approval of this research<br>• Indicate the form of consent obtained (written/oral) or the reason that consent was not obtained (e.g. the data were analyzed anonymously)<br><br>**Animal Research (involving vertebrate animals, embryos or tissues)**<br>• Provide the name of the Institutional Animal Care and Use Committee (IACUC) or other relevant ethics board that reviewed the study protocol, and indicate whether they approved this research or granted a formal waiver of ethical approval<br>• Include an approval number if one was obtained<br>• If the study involved *non-human primates*, add *additional details* about animal welfare and steps taken to ameliorate suffering<br>• If anesthesia, euthanasia, or any kind of animal sacrifice is part of the study, include briefly which substances and/or methods were applied<br><br>**Field Research**<br><br>Include the following details if this study involves the collection of plant, animal, or other materials from a natural setting:<br>• Field permit number<br>• Name of the institution or relevant body that granted permission	
**Data Availability**<br><br>Authors are required to make all data underlying the findings described fully available, without restriction, and from the time of publication. PLOS allows rare exceptions to address legal and ethical concerns. See the PLOS Data Policy and FAQ for detailed information.	Yes - all data are fully available without restriction

A Data Availability Statement describing where the data can be found is required at submission. Your answers to this question constitute the Data Availability Statement and **will be published in the article**, if accepted.<br><br>**Important:** Stating 'data available on request from the author' is not sufficient. If your data are only available upon request, select 'No' for the first question and explain your exceptional situation in the text box.<br><br>Do the authors confirm that all data underlying the findings described in their manuscript are fully available without restriction?	
**Describe where the data may be found in full sentences. If you are copying our sample text, replace any instances of XXX with the appropriate details.**<br><br>• If the data are **held or will be held in a public repository**, include URLs, accession numbers or DOIs. If this information will only be available after acceptance, indicate this by ticking the box below. For example: *All XXX files are available from the XXX database (accession number(s) XXX, XXX.*).<br>• If the data are all contained **within the manuscript and/or Supporting Information files**, enter the following: *All relevant data are within the manuscript and its Supporting Information files.*<br>• If neither of these applies but you are able to provide **details of access elsewhere**, with or without limitations, please do so. For example:<br><br>*Data cannot be shared publicly because of [XXX]. Data are available from the XXX Institutional Data Access / Ethics Committee (contact via XXX) for researchers who meet the criteria for access to confidential data.*<br><br>*The data underlying the results presented in the study are available from (include the name of the third party*	All relevant data are within the manuscript and its Supporting Information files.

Powered by Editorial Manager® and ProduXion Manager® from Aries Systems Corporation

and contact information or URL).<br>• This text is appropriate if the data are owned by a third party and authors do not have permission to share the data.<br><br>* typeset	
Additional data availability information:	

Manuscript
Click here to access/download;Manuscript;Manuscript.docx

The role of information communication technologies as a moderator of knowledge creation and knowledge transfer in improving the quality of healthcare services

(Short title: ICT as a moderator of knowledge creation and transfer for improved healthcare quality)

Simon Colnar[1], Ivan Radević[2*], Nikola Martinović[2], Anđelko Lojpur[2], Vlado Dimovski[1]

[1] University of Ljubljana, School of Economics and Business, Kardeljeva ploščad 17, 1000 Ljubljana
[2] University of Montenegro, Faculty of Economics, Ul. Jovana Tomaševića br. 37, 81000 Podgorica

* Corresponding author:
Tel: +382 67 629 888; E-mail: radevic@ucg.ac.me

These authors contributed equally to this work.

Abstract:

This study examines the role of knowledge creation, knowledge transfer and information communication technologies, which are organizational factors that influence the quality of healthcare services. In today's knowledge-intensive environment, understanding and gaining in-depth knowledge on how to improve the quality of healthcare services is gaining in importance and recognition. Quantitative data collected in 2019 with 151 respondents employed in healthcare organizations was used. Running a series of hierarchical linear regression models, we found a significant positive relationship between knowledge creation and quality of healthcare services, and a significant positive relationship between knowledge transfer and quality of healthcare services. Empirical data additionally provides support for information communication

technologies that act as a moderator both in the relationship between knowledge creation and knowledge transfer with quality of healthcare services. With our data, we provide empirical backing for the impact of knowledge creation, knowledge transfer and information communication technologies on the quality of healthcare services that are provided by Montenegrin healthcare organizations. Our paper offers theoretical and practical implications derived from our research study.

Keywords: quality of healthcare services, knowledge management, information and communication technologies, knowledge creation, knowledge transfer.

1. Introduction

Information and communication technologies are identified as one of the crucial enablers of knowledge management practices and most relevant and contemporary literature suggests that appropriate technology solutions within organizations are of significant importance in relation to successful knowledge management initiatives [1]. In today's knowledge intensive world of work [2], the concept of knowledge management is becoming increasingly important as a tool that may be vital to a higher level of organizational effectiveness. Ongoing growing importance of information communication technologies has already changed traditional forms of organizational functioning, which consequently determined the concept of knowledge management to become integral as a tool for achieving higher levels of organizational effectiveness [3]. This change in terms of the approach paradigm, emphasizes even more the role of knowledge as a determining factor of improved organizational performance [4]. In essence, this concept implies a process of efficient and effective learning, through research, exploitation and sharing of human knowledge, with a support of adequate technological advancements [5]. Previous research posits the concept

of knowledge management as a determinant of organizational success [6], improved service quality [7] and as a tool that enables organizations to make internal improvements [8, 9].

Nowadays, the field of healthcare services is continuously exposed to pressures from different stakeholders to improve the quality of its services [10]. Moreover, researchers Parand et al. [11] suggest that a number of challenges related to quality of healthcare services remain unsolved and require the attention of both academics and practitioners, especially as costs associated with healthcare will continue to rise in the future [12]. Therefore, it becomes crucial to gain in-depth knowledge and understanding regarding healthcare service quality dimensions and define actions that could help healthcare services providers with improving their overall organizational effectiveness [13]. There is a wide range of industries, where the application of knowledge management can result in positive improvements of organizational performance, including healthcare services, where the knowledge of employees represents the core of providing care for patients. Moreover, an adequate knowledge management process results in the adoption of quality decisions by healthcare professionals, and in better outcomes for patients [14]. In addition, the adequate knowledge management process in healthcare organizations is also vital for raising the level of healthcare services in practice [15]. With our paper, we aim to respond to calls of researchers to enhance the knowledge regarding the concept of quality of healthcare services [16] and its subsequent improvement [17] and to gain additional understanding of knowledge management as applied in the healthcare environment [18].

In current state-of-the-art research there is a gap in considering the impact of specific organizational factors such as knowledge creation, knowledge transfer and information communication technologies on the quality of healthcare services. Additional insight is of paramount importance as it enables healthcare services providers to gain knowledge about

potential activities and solutions for improving the quality of healthcare services [11]. As existing theory seems to be difficult to apply within the healthcare environment, we focus within the scope of our paper on improving the understanding and gaining further knowledge of the construct of knowledge creation, where researchers [19] claim that knowledge creation leads to improved organizational performance, which applies also to public sector organizations, including healthcare institutions. In a similar vein, we intend to strengthen previous research by providing further insight into the impact of knowledge transfer on achieving higher levels of organizational performance [20], which is in this paper explored as the quality of healthcare services. Previous research validates the positive relationship between information communication technologies and enhanced organizational performance [21], where we aim to provide additional insight into information communication technologies and their impact on improving the quality of healthcare services.

The purpose of our paper is to add to contemporary research, by theoretically proposing a conceptual model and empirically testing the impact of organizational factors that influence knowledge management activities within the healthcare sector as part of the ongoing attempts to enhance the quality of healthcare services [22]. We investigate the relationship between knowledge creation and quality of healthcare services and knowledge transfer and quality of healthcare services. In addition, we explored for the potential moderating effect of information communication technologies on the aforementioned proposed relationships. We utilized a quantitative analysis of collected data from healthcare employees to test our hypotheses in healthcare institutions in Montenegro. As we obtained data for all our variables in a one-time single survey, we acknowledge that common method bias might be a methodological issue in our study. With our paper, we aim to contribute towards advancing the body of literature of quality of

healthcare services and knowledge management in the context of healthcare organizations [23], where currently a gap in knowledge exists [16, 24]. In line with our overarching theory of the knowledge-based view of the organization [25, 26, 27], we emphasize the important role of knowledge in healthcare organizations as we propose that knowledge management is one of the primary sources that influence the functioning of such organizations and subsequently has the potential to increase the quality of healthcare services that are offered to users in practice. Previous research within the knowledge-based view of the organization [25, 28] suggests that the presence and right utilization of knowledge has the potential to lead to higher levels of organizational performance [29]. Similarly, contemporary research of Martin and Javalgi [30] posits that the attainment and deployment of knowledge is integral for organizational performance. In addition, we aim to empirically clarify the relationship between organizational factors that impact knowledge management and its relationship with organizational performance [31].

2. Literature Review

Healthcare systems, as well as micro-level health facilities, generally depend on data and information collected by patients, medical doctors, or obtained from scientific studies [32, 33]. In this context, management of information, knowledge creation and knowledge transfer, are key areas in the healthcare system. An improved knowledge management system contributes to better decisions of healthcare professionals and results in better treatment outcomes as a result of the healthcare provided [34]. The extent to which knowledge creation and knowledge transfer contribute to better treatment outcomes is one of the key information that we seek to obtain. Efforts for quality improvements in healthcare are interdisciplinary as they often involve various fields such as health service (knowledge) management, public health, healthcare professionalism and health service design [35]. In addition, healthcare services are continuously evolving and changing

as new scientific knowledge emerges and contemporary technologies become available [36], which consequently impacts the quality of healthcare services that are provided in practice.

2.1. Knowledge Creation and Quality of Healthcare Services

Knowledge creation is a continuous process that implies ongoing interaction between individuals and groups at the organizational level [37, 38, 39]. Moreover, knowledge creation as a process consists of four stages: socialization, externalization, combination, and internationalization [40]. Creation of new knowledge can similarly be the basic source of competitive advantage over longer periods of time [41] and is useful for any organization, whether public or private. Creating a comprehensive system that enhances the process of creating new knowledge, helps the organization achieve its strategic goals [26]. In the area of healthcare services, the strategic goal is higher quality of services provided, through a combination of efforts of healthcare professionals and direct interaction with patients [42]. Knowledge creation and its inter-organizational dissemination, through the use of an adequate network concept [43] and the necessary data cataloguing, contributes to positive repercussions on the organizational performance of healthcare providers. Since knowledge creation is a continuous process, its constant improvement is vital for the benefit of all stakeholders. Knowledge creation can have a significant positive impact on professional development of employees in the environment of healthcare institutions [44]. In addition, modern development of technology is a significant accelerator of the process of knowledge creation at the organizational level [45, 46]. Consequently, this combination of the healthcare system and information communication technologies has changed the way healthcare is provided and it contributed to greater benefits for patients [47]. In spite of the existence of solid scientific research, aimed at analysing the relationship between the concept of knowledge creation and organizational performance [48], with the influence of information communication

technologies as a newly associated scalar value [49], the subject area has not been analysed adequately within the concept of quality of healthcare services in the healthcare system of Montenegro. This paper, based on empirical research, aims to close this gap. In view of this, our first hypothesis is:

Hypothesis 1: Knowledge creation is positively related to the quality of healthcare services

2.2. Knowledge Transfer and Quality of Healthcare Services

Knowledge transfer in organizations is defined as the process through which individuals, groups, departments or the whole organization are affected by the experience and knowledge of another [50]. Knowledge transfer within healthcare organizations is recognized as one of the main indicators of quality, innovation, competitiveness, growth and development of the organization. The importance of the impact of knowledge transfer on the quality of healthcare services has caused the recent growth of interest in the area [51]. Existing studies similarly identified quality information exchange as one of the key indicators of quality of services within healthcare organizations in addition to the competences of healthcare professionals [52, 53, 54]. Moreover, cross-national studies have indicated the importance of organizational culture as a component that influences the willingness of health professionals to be active participants in the process of knowledge transfer [55]. A system that is too centralized negatively affects the process of knowledge transfer at the organizational level [56], so it is necessary to find the right balance also in this aspect. The existence of an optimal level of theoretical knowledge and practical skills and competences of healthcare professionals are key indicators of successful knowledge transfer among healthcare professionals [55]. Improving the quality of healthcare services is ever more based on the improvement of both knowledge creation and knowledge transfer [57]. In addition,

globally there is a growing social, political and social interest in the exchange of knowledge and experience in the context of improving healthcare, while emphasizing the key role of scientific community and medical staff in the process of generating new value and new knowledge [58]. Based on previous research, our paper further deepens the analysis of the subject area through the study of knowledge transfer and the quality of healthcare services within the Montenegrin healthcare system. The second hypothesis arises from the above:

Hypothesis 2: Knowledge transfer is positively related to the quality of healthcare services

2.3. Information Communication Technologies and Quality of Healthcare Services

Information communication technologies, through the use of computers, the Internet, mobile devices and various interactive platforms, significantly shape the functioning of modern organizations, their systems, processes and communication [59]. Advanced information communication technologies help the process of knowledge creation through a number of different functionalities such as analysis and presentation, data storage and management, networking and communication, as well as interaction and collaboration [60, 61, 62, 46]. Creating new knowledge and innovation has become crucial in the process of implementing information communication technologies in the regular practice of healthcare institutions [63]. The creation of information and creation of new knowledge is the area where information communication technologies, may contribute to a higher quality of healthcare services [64]. Nowadays, the ability to effectively access needed information and to distinguish between relevant and irrelevant information is becoming an ever important skill for professionals and organizations [65]. In this context, Soto-Acosta and Cegarra-Navarro [66] emphasize the role of information communication technologies in the exploitation and management of existing knowledge. The ability of individuals,

professionals and organizations to have access to and later to disseminate health related information in today’s electronic society emphasizes the necessity of adopting information communication technologies [67] within the healthcare environment. Additionally, this possibility for creating new knowledge, through information communication technologies, enables the application of the concept "*patient-centered care*", which implies directly and positively a higher quality of services provided [68]. Andreeva and Kianto [1] also point to the specific role of information communication solutions at the level of organizational knowledge, organizational performance, and organizational competitiveness. In this paper, we tested the concept of improving the quality of healthcare services, through the moderating effect of information communication technologies, defining the third hypothesis as follows:

Hypothesis 3: Information communication technologies moderate the positive relationship between knowledge creation and quality of healthcare services

In addition to knowledge creation, knowledge transfer is an area where information communication technologies contribute to the sharing of good practice in healthcare. Information communication technology platforms, in various forms and shapes, enable the knowledge transfer among healthcare professionals [69]. The appropriate utilization of information communication technologies contributes to faster and better inter-organizational knowledge transfer, all to the benefit of patients as end users [70]. The application of various tools and applications enables a quicker transfer of knowledge both between service providers and between providers and users of healthcare services, thus raising awareness of the importance of healthcare [71]. This can play a very important role also in providing remote healthcare, through the use of technology and rapid exchange of information related to the medical state of patients, implementation of adequate diagnostics, treatments and disease remediation [72]. We tested empirically the understanding of

the moderating effect of information communication technologies in the relationship between knowledge transfer and quality of healthcare services, by setting the following hypothesis:

Hypothesis 4: Information communication technologies moderate the positive relationship between knowledge transfer and quality of healthcare services

We present our conceptual model with the above mentioned hypotheses in Fig 1.

Fig 1. Conceptual model of the relationships between knowledge creation, knowledge transfer, quality of healthcare services and information communication technologies.

3. Methodological approach

3.1. Data Collection Procedure and Measurement

The process of collecting primary data involved the use of a questionnaire to understand the process of knowledge creation, knowledge transfer and information communication technologies within healthcare organizations with a special focus on the quality of services provided. A sample of 45 health care institutions in Montenegro was generated by a combination of institutions from public (32) and private sector (13).

The data were collected in May 2019, and 151 healthcare workers took part in the survey. In order to ensure the principles of impartiality and non-selectivity and the concept of comprehensiveness, information was collected across various organizational levels in healthcare institutions. Accordingly, the sample consists of 45 directors, 45 medical doctors, 45 technicians, and 16

members of the Union of Medical Doctors, which altogether makes a total of 151 individuals whose opinion was taken into account. Just over three-fifths of the sample (60.3%) are women, and the remaining two-fifths (39[illegible]7%) are men. When it comes to age structure, respondents aged between 50 and 59 are a dominant portion of the sample (37.5%), followed by respondents aged 30 and 39 (24.3%), and respondents aged 40 and 49 (20.1%).

Majority of healthcare workers, 94.7%, have worked in the healthcare system for more than five years, 93.3% have worked for more than five years for their current employer (i.e. medical organization), hence the sample is representative in terms of the respondents' ability to realistically perceive the processes of knowledge creation and knowledge transfer, information communication technologies and quality of healthcare services in the context of analysed variables of this study.

To analyse if our results might be biased, we applied Harman’s one-factor test [73]. The obtained results indicate that the first factor makes 56.7% of the total variance. This result is slightly above the recommended value threshold (50%) by Podsakoff et al. [74], suggesting that common method bias might be a limiting factor in the study. The obtained data were imported within the SPSS 25.0 version.

In order to analyse each individual research construct, the authors used measuring instruments that have a high frequency of use and are adequately validated in contemporary scientific research. All measuring instruments meet a predefined set of criteria: they are often cited in research papers published in relevant scientific journals, they are up-to-date in the sense that they are used in the most recent research, and finally, they are well conceptually established in the context of their frequent use by key authors from our research scope.

Knowledge Creation: Two item scale was used to determine this construct (α =.90) [75, 76]. The measurement of this variable was conducted by measuring the degree of agreement of the respondents with the following items: "*My organization has mechanisms for creating or acquiring knowledge from different sources such as volunteers, clients, donors or competitors*".

Knowledge Transfer: This construct was observed through the use of an eight item scale (a =.93) [75, 77]. The questionnaire involves answering items such as "*In my organization, it is easy to identify key experts in certain areas and learn how to get in touch with them*".

Quality of Healthcare Services: Measured with a scale that consists of 3 items (α =.87) [78]. The questionnaire consists of the following items: "*Within my organization, we provide higher quality services to our customers*" and "*All in all, our organization works better*".

Information Communication Technologies: A two item scale was used in this case (α =. 67) [79]. It is based on statements examining whether technical support to employees is always available, as well as whether employees are confident enough to use information communication technologies or avoid using them due to lack of experience. The questionnaire comprises of items such as: *"Technical support for information systems is readily available"*.

Control variables: As for control variables, there are two control variables that make an integral part of our research: age and the highest level of education. In research, the decision to include or exclude control variables may have implications for drawing final conclusions based on the research conducted [80]. Against the background of the individual and existing knowledge management research, demographic characteristics, such as age and the highest level of education, may have an impact on the overall level of knowledge management activities in an organization, which is the subject of analysis [81]. It is important to note that both control variables that are

considered control in our research have already been the subject of analyses in researches that covered knowledge management [82].

3.2. Methods

To explore the convergent validity of all items utilized to measure constructs in our research we examined standardized factor loadings [83]. In Table 1, we report the range of our standardized factor loadings in our measurement model. Standardized factor loadings for all of our four constructs were statistically significant (> .50). One item intended to measure knowledge creation and two items intended to measure quality of healthcare services and information communication technologies did not meet the criteria recommended in the literature and were therefore omitted from the final model. Our final model consists of 15 items utilized to evaluate the existing state of four measured constructs. To test the composite (constructs) reliability we explored the composite reliability index (hereinafter: CRI) and average variance extracted (hereinafter: AVE) [84]. To fulfill research criteria, we follow the suggested values of Diamantopolous and Sigaw [85], which are for AVE (.40) and CRI (.60). We present AVE and CRI values for our measured constructs in Table 1. Numerous fit indices to evaluate the model fit to data at the global level exist [86]. Fit indices are as follows: CFI = 0.97; chi-square: 104.690; RMSEA = .07; and df = 67* and are satisfactory.

Table 1: Confirmatory factor analysis results

Construct	No. of items	Reliability (Cronbach alpha)	Range of standardized coefficients (factor loadings)	CRI	AVE
Knowledge Creation	2	0.90	0.82 to 0.86	0.83	0.71

* Without modification indices, the results of the model fit were: CFI = .84, chi-square = 296.722, RMSEA = .14, and df = 84.

Knowledge Transfer	8	0.93	0.65 to 0.82	0.90	0.54
Quality of Healthcare Services	3	0.87	0.64 to 0.92	0.83	0.62
Information Communication Technologies	2	0.67	0.72 to 0.84	0.76	0.61

4. Results

Selected descriptive statistics for our measured variables are presented in Table 2. Respondents on average value quality of healthcare services (4.06) the best in their organization, followed by knowledge transfer (3.90) and knowledge creation (3.71). The construct of information communication technologies received a significantly lower evaluation (2.40). Between our measured variables the correlation coefficients are moderately or strongly positive with ranges between .65 and .88 and moderately or weakly negative with ranges between -.18 and -.28. A significant and positive correlation was evident between knowledge transfer and quality of healthcare services (.71; $p < 0.01$) and knowledge transfer and knowledge creation (.88; $p < 0.01$). In addition, there was a significant and negative correlation between knowledge transfer and information communication technologies (-.28; $p < 0.05$). Quality of healthcare services displayed a significant and positive correlation with knowledge creation (.65; $p < 0.01$) and a significant and negative correlation with information communication technologies. In the scope of our research, knowledge creation had a significant and negative correlation with information communication technologies (-.25; $p < 0.01$). Between our two control variables there is no significant correlation.

Table 2: Mean Values, Standard Deviations and Coefficient Correlations (n=151).

Variable	Mean	SD	1	2	3	4	5

1. Age	48.82	10.76					
2. Highest Level of Education	4.72	0.64	-0.06				
3. Knowledge Transfer	3.90	0.94	0.11	0.01			
4. Quality of Healthcare Services	4.06	0.86	-0.01	-0.05	0.71**		
5. Knowledge Creation	3.71	1.10	0.05	-0.01	0.88**	0.65**	
6. Information Communication Technologies	2.40	0.85	-0.01	-0.12	-0.28**	-0.18*	-0.25**

**$p < 0.01$ and *$p < 0.05$

Legend: SD = Standard Deviation, p = significance

Hypothesis 1 (H1) explored the direct relationship between knowledge creation and quality of healthcare services. Hypothesis 2 (H2) examined the direct relationship between knowledge transfer and quality of healthcare services. In hypothesis 3 (H3), we include information communication technologies as a moderator of the relationship between knowledge creation and quality of healthcare services. Similarly, in hypothesis 4 (H4) we include information communication technologies as a moderator of the relationship between knowledge transfer and quality of healthcare services. We ran a series of hierarchical regression analysis utilizing centered variables to test our conceptual model that is explained with our proposed hypotheses in Fig 1. In our first direct effect model (model 1), we include knowledge creation as the independent variable and age and highest level of education as our control variables. Within model 2, we include knowledge transfer as the independent variable and the aforementioned control variables. In addition, in our third model (model 3) we explore the suggested two-way interaction effect between knowledge creation and information communication technologies. In model 4, we include our second proposed interaction effect between knowledge transfer and information communication technologies. We present a more in-depth analysis of our four models in Table 3.

Table 3: Hierarchical regression analysis predicting quality of healthcare services – Models 1-4.

Variables	Model 1				Model 2				Model 3				Model 4			
	b	s.e.	ß	t	b	s.e.	ß	t	b	s.e.	ß	t	b	s.e.	ß	t
Age	-.004	.005	-.055	-.766	-.002	.005	-.028	-.412	-.004	.005	-.048	-.685	-.002	.005	-.029	-445
Highest Level of Education	-.166	.091	-.132	-1.817	-.161	.086	-.128	-1.869	-.165	.089	-.131	-1.848	-.177	.083	-.141	-2.131*
C_KNOC	.471	.057	.604	8.258**					.464	.056	.595	8.344**				
C_KNOT					.560	.059	.658	9.538**					.542	.057	.637	9.561**
C_ICT	-.071	.127	-.041	-.556	-.092	.121	-.053	-.764	.084	.137	.049	.616	.083	.127	.048	.650
C_KNOCxC_ICT									.242	.090	.208	2.699**				
C_KNOTxC_ICT													.304	.091	.240	3.339**
R^2	0.369				0.436				0.404				0.483			
F(df)	17.96(123)				23.76(123)				6.61(122)				22.81(122)			
ΔR^2	0.369				0.436				0.035				0.047			

*p < 0.05

**p < 0.01

We found a significant and positive relationship in model 1, between knowledge creation (β = .60; exact p = .000) and quality of healthcare services. We are able to provide empirical support for H1 with our data. In addition, we also found a significant and positive relationship between knowledge transfer (β = .66; exact p = .000) and quality of healthcare services in model 2. Therefore, we are also able to provide empirical support for H2 on the basis of our data. In models 3 and 4 we included information communication technologies as the moderator of knowledge creation (model 3) and knowledge transfer (model 4) with quality of healthcare services. Both models showed considerable added value in relation to the direct effect models as expressed in model 1 and 2. The R^2 change is .035 in model 3 in comparison with model 1 and .047 in model 4 in comparison with model 2.

Our results in model 3 show a significant and positive relationship on the example of our two-way interaction effect between knowledge creation and information communication technologies and on the quality of healthcare services (β = .21; exact p = .008). In line with the above, we are able

to provide empirical support also for our H3. Similarly, model 4 indicates a significant and positive relationship of our two-way interaction effect consisting of knowledge transfer and information communication technologies with quality of healthcare services ($\beta = .24$; exact $p = .001$). With our results, we are to support H4 on the basis of empirical data. Additionally, we present the simple slope analysis of both H3 and H4. The simple slope analysis for H3 indicates it is significant (exact $p = .000$). Moreover, we present the interaction between knowledge creation and information communication technologies and their influence on quality of healthcare service (see Fig 2).

Fig 2. Interaction between knowledge creation and information communication technologies in influencing quality of healthcare services.

Highest levels of quality of healthcare services occur when the level of information communication technologies is high. In addition, the impact of knowledge creation is similarly important as both in the case of low information communication technologies and high information communication technologies, higher levels of knowledge creation indicate to better quality of healthcare services. In the example of high knowledge creation, best quality of healthcare services is also related to high information communication technologies, while the comparison to low information communication technologies shows a significant difference. In the example of low knowledge creation, low information communication technologies imply better quality of healthcare services.

Moreover, the analysis of the simple slope for H4 is also significant (exact $p = .000$). The graphical representation of the interaction effect between knowledge transfer and information

communication technologies as they influence the quality of healthcare services is presented in Fig 3.

Fig 3. Interaction between knowledge transfer and information communication technologies in influencing quality of healthcare services.

The highest levels of quality of healthcare services are related to high levels of information communication technologies. Moreover, also the influence of knowledge transfer is important as higher levels of knowledge transfer positively influence the quality of healthcare services. When levels of knowledge transfer are low, low information communication technologies contribute to better quality of healthcare services. Interestingly, when levels of knowledge transfer are low, the combination with higher levels of information communication technologies produces worse results in terms of quality of healthcare services.

5. Discussion

This paper involved structuring a conceptual research model that is composed of four constructs: knowledge creation, knowledge transfer, information communication technologies and the level of quality of provided healthcare services. The study implied setting up of four hypotheses in order to analyse the relationship between the defined variables, through the implementation of a hierarchical linear regression model. The first hypothesis starts from the assumption that knowledge creation is in a direct positive correlation with the level of quality of healthcare services. The second hypothesis also implies a positive correlation between knowledge transfer

and the quality of healthcare services, while the third and fourth examine the moderating role that information communication technologies play in fostering knowledge creation and knowledge transfer that lead to better performance of healthcare providers.

The results of the conducted research confirm the existence of a direct positive correlation between knowledge creation and the quality of healthcare services (H1). In other words, knowledge creation is vital in determining the quality of healthcare provided to patients. Such results are in accordance with previous research, which argued that knowledge creation leads to improved organizational performance [19]. Similarly, our results further validate the findings of Ayanbode and Nwagwu [44] and Kitson et al. [43], that knowledge creation has a positive impact on the organizational performance also on the example of healthcare organizations.

The results obtained on the basis of the study confirmed the second hypothesis, the existence of a positive correlation between knowledge transfer and the level of quality of healthcare services provided (H2). Knowledge transfer enables the dissemination of best medical practices and a better output in terms of healthcare provided to patients. Even in situations where it is applicable, the valuable knowledge gained from medical research is essentially useful only if used by all stakeholders within the healthcare system. Unfortunately, healthcare providers in practice often lack information about current trends, latest scientific knowledge and researches that applied best medical practice, and are not aware how their application could be a deciding factor in treatment of patients. Under such circumstances, patients are hindered as they do not receive the best possible treatment, or receive the one that does not match entirely their specific health condition [87]. Therefore, the transfer of newly created knowledge and medical practice is an important determinant of healthcare provided. Our results support the claim of Lombardi [20] that knowledge transfer influences the achievement of higher levels of organizational performance. In a similar

vein, our results are in line with the suggestions of Wensing and Grol [57] that knowledge creation has an important impact on improving the quality of healthcare services.

The results of our conducted research provided empirical support to the third hypothesis. Information communication technologies moderate the positive correlation between knowledge creation and the level of quality of healthcare services (H3). With our results we are able to further validate the opinion of Tripathi et al. [64] that information communication technologies act as a moderator in the relationship between knowledge creation and higher quality of healthcare services. Similarly, we add to state-of-the-art research of Papanastasiou et al. [68] and Andreeva and Kianto [1] that emphasize the specific role of information communication technologies on the improvement of healthcare services.

Finally, this study confirmed that information communication technologies also play an important role in facilitating the transfer of knowledge, and that they contribute to a higher level of quality of healthcare services (H4). The higher level of knowledge transfer is closely related to the quality of healthcare services, and information communication technologies are the factor that is vital to the progress in this field. Such findings correlate to existing literature that stresses the impact of information communication technologies on knowledge transfer [69] and its subsequent influence on improving the quality of healthcare services [70].

Information communication technologies enable the knowledge transfer and the dissemination of good practices at various different organizational levels. Universities and research institutes remain the main hubs of sources for the creation and transfer of newly created knowledge in the field of healthcare. The use of information communication technologies and documenting best practices in healthcare institutions requires constant work on improving and updating the whole

system. This can be a challenge, given the speed of technological change and the growing need for rapid dissemination of new knowledge, especially in times of crisis, such as the current global COVID-19 pandemic. The organizational goal must be to create a system that will allow medical doctors and other healthcare professionals to know at all times where to find up-to-date information related to a specific field of medicine, or to access newly available knowledge [88].

There are several theoretical contributions of our research. First, with our results we contribute to existing research that focuses on the knowledge-based view of the organization [25, 26, 27] as we emphasize the integral role of knowledge within the environment of healthcare organizations. Furthermore, we add to up-to-date research by theoretically proposing a conceptual model and empirically testing organizational factors of knowledge management that have the potential to increase the quality of healthcare services [22]. By testing and validating our model on primary data, we are able to contribute to a broader understanding and further insight on knowledge and knowledge management [25, 28] and the positive influence on organizational performance [29, 30]. Second, with our research we respond to some of the existing challenges that are related to the area of quality of healthcare services [11] as we provide empirical insight into actions that could help services providers with improving quality of their healthcare services as was suggested in Tripathi and Siddiqui [13]. Third, we add to the debate of knowledge creation and its influence on the advancement of organizational performance [19, 43, 44]. With our research, we extend the understanding of the aforementioned relationship on the example of healthcare institutions. Fourth, we provide additional support to suggestions that knowledge transfer has a positive impact on improving the overall organizational performance [20] in our case understood as the quality of healthcare services. With this part of our research, we validate the opinion of Wensing and Grol [57] that improving the quality of healthcare services is also related to the levels of knowledge

transfer within an organization. Fifth, with our results we are able to promote the positive impact of information communication technologies on improved organizational performance as was emphasized in the research of Yunis et al. [21]. In addition, our results are in line with Tripathi et al. [64], where the authors suggest that information communication technologies as a moderator can contribute towards achieving higher levels of quality of healthcare services in practice. Sixth, with our study we are able to extend the understanding of knowledge management in the context of the public sector [23, 24], where we focused our research in exploring knowledge management within the healthcare environment, which was typically overlooked in previous studies.

The practical implications of our research are intended for managers, practitioners and decision makers and are identified as opportunities for improvements within the healthcare system in Montenegro, through better understanding and knowledge of organizational factors such as knowledge creation, knowledge transfer and information communication technologies. Those organizational factors are instruments that can positively influence the levels of quality of healthcare services. The content and suggestions that can be derived from our research in the form of concrete recommendations can help stakeholders engaged in the healthcare system to create appropriate conditions for achieving better organizational performance, which will subsequently raise the quality of healthcare services. Hopefully, on the basis of our results, managers and employees in healthcare organizations will devote more attention, resources and efforts towards implementing activities and initiatives that include knowledge creation, knowledge transfer and information communication technologies with the final aim of improving the overall quality of healthcare services. Relying on empirical evidence, our study offers the opportunity or starting point for interested stakeholders to improve their knowledge, skills and competences related to knowledge management and providing quality healthcare services in practice. Nevertheless, it is

important that managers are aware of the relevant alignment of their knowledge management activities within their specific organizational context and also in relation to their available knowledge management resources. In addition, within the specific and complex healthcare environment, identified and assessing appropriate knowledge management activities might be even more challenging.

In spite of the numerous theoretical and practical contributions of our paper, some limitations exist. First, the methodological issue of common method bias as revealed by Harman's single factor test [73] is present. Second, as our results are based on a sample from only one country, we argue that it would be beneficial to conduct a cross-national study to provide a higher degree of generalization of our findings. Third, we have to take into account the complexity of the healthcare environment, which might negatively influence the responses from our respondents as they are constantly exposed to demanding and draining situations at work. Fourth, within this research we did not distinguish between some of the factors determining the characteristics of healthcare organizations such as the size of the organization that could be measured with the number of its employees. Fifth, in general in the healthcare environment, measuring the quality of healthcare services is difficult as we are predominantly relying on the perception of either healthcare services providers or healthcare services users.

Given the limitations of our study, we identified additional opportunities for future research on the topic of quality of healthcare services, including the following: (1) to counteract the potential negative effect of common method bias, we propose to collect the data for the dependent, independent and moderating variables at different points in time; (2) we would advise to focus on conducting similar research on an international sample; (3) to mitigate the negative effect of potential employee bias, we should include a higher number of respondents in future studies; (4)

future research should include additional control variables that are measured at the organizational level; (5) it is necessary to promote international efforts to standardize the measurement of quality of healthcare services, which would additionally enable the comparison between different countries.

6. Conclusion

The proposed conceptual model and the conducted empirical study within our research is aimed at examining the impact of knowledge creation and knowledge transfer on the level of quality of provided healthcare services. Thus, our study contributes towards the expansion of the conceptual framework of knowledge management within the healthcare environment and aims to support ongoing efforts to improve the overall quality of healthcare services in practice. More specifically, we explored the moderating effect that information communication technologies might have on the existence of the two predefined relationships. Obtained results clearly indicate a direct and positive link between knowledge creation and quality of healthcare services and between knowledge transfer and quality of healthcare services. Moreover, information communication technologies further moderate the relationships that are important in influencing the quality of healthcare services. Highest levels of quality of healthcare services occur when the level of information communication technologies is high. In a nutshell, the impact of knowledge creation and knowledge transfer is important as higher levels of knowledge creation and knowledge transfer lead to better quality of healthcare services. Additionally, our study proposes some promising directions to conduct future research on this topic.

REFERENCES

1. Andreeva T, Kianto A. Does knowledge management really matter? Linking knowledge management practices, competitiveness and economic performance. Journal of knowledge management. 2012; 16: 617–636. https://doi.org/10.1108/13673271211246185.
2. Cross Walker T. Inclusive talent management in the public sector: theory and practice. Transnational Corporations Review. 2020; 12: 140–148. https://doi.org/10.1080/19186444.2020.1741296.
3. Raudeliūnienė J, Davidavičienė V, Jakubavičius A. Knowledge management process model. Entrepreneurship and Sustainability Issues. 2018; 5: 542–554. https://doi.org/10.9770/jesi.2018.5.3(10).
4. Tarango J, Machin-Mastromatteo JD. The Role of Information Professionals in the Knowledge Economy: Skills, Profile and a Model for Supporting Scientific Production and Communication. Oxford: Chandos Publishing; 2017.
5. Jashapara A. Knowledge management: an integrated approach. London: Pearson Education; 2011.
6. Baldé M, Ferreira AI, Maynard T. SECI driven creativity: the role of team trust and intrinsic motivation. Journal of Knowledge Management. 2018; 22: 1688–1711. https://doi.org/10.1108/JKM-06-2017-0241.
7. Colnar S, Dimovski V, Bogataj D. Knowledge Management and the Sustainable Development of Social Work. Sustainability. 2019; 11: 6374. https://doi.org/10.3390/su11226374.
8. Dimovski V, Penger S, Peterlin J, Grah B, Černe M, Klepec M. Advanced Management and Leadership Practice. Essex: Pearson Education Limited; 2017.
9. Lojpur SA, Radević I, Lojpur A, Martinović N. Is the Recovery of Transition Countries Possible on the New Global Stage and How? International Journal of Innovation and Economic Development. 2020; 6: 19–30. https://doi.org/10.18775/ijied.1849-7551-7020.2015.61.2002.
10. Al-Borie HM, Sheikh Damanhouri AM. Patients' satisfaction of service quality in Saudi hospitals: a SERVQUAL analysis. International Journal of Health Care Quality Assurance. 2013; 26: 20–30. https://doi.org/10.1108/09526861311288613.
11. Parand A, Dopson S, Renz A, Vincent C. The role of hospital managers in quality and patient safety: A systematic review. BMJ Open. 2014; 4: 1–15. https://doi.org/10.1136/bmjopen-2014-005055.
12. Hall A. Quality of Life and Value Assessment in Health Care. Health Care Analysis. 2020; 28: 45–61. https://doi.org/10.1007/s10728-019-00382-w.
13. Tripathi SN, Siddiqui MH. Assessing the quality of healthcare services: A SERVQUAL approach. International Journal of Healthcare Management. 2018; 13: 133–144. https://doi.org/10.1080/20479700.2018.1469212.
14. Jahmani K, Fadiya SO, Abubakar AM, Elrehail H. Knowledge content quality, perceived usefulness, KMS use for sharing and retrieval: A flock leadership application. VINE

Journal of Information and Knowledge Management Systems. 2018; 48: 470–490. https://doi.org/10.1108/VJIKMS-08-2017-0054.

15. Shahmoradi L, Safadari R, Jimma W. Knowledge Management Implementation and the Tools Utilized in Healthcare for Evidence-Based Decision Making: A Systematic Review. Ethiopian journal of health sciences. 2017; 27: 541–558. https://doi.org/10.4314/ejhs.v27i5.13.
16. Klemenc-Ketiš Z, Švab I, Poplas Susič A. Implementing quality indicators for diabetes and hypertension in family medicine in Slovenia. Zdravstveno Varstvo. 2017; 56: 211–219. https://doi.org/10.1515/sjph-2017-0029.
17. Junghans T. Don't Mind the Gap!" Reflections on Improvement Science as a Paradigm. Health Care Analysis. 2018; 26: 124–139. https://doi.org/10.1007/s10728-017-0353-7.
18. Han J, Pashouwers R. Willingness to share knowledge in healthcare organisations: The role of relational perception. Knowledge Management Research and Practice. 2018; 16: 42–50. https://doi.org/10.1080/14778238.2017.1405144.
19. Boon Sin A, Zailani S, Iranmanesh M, Ramayah T. Structural equation modelling on knowledge creation in Six Sigma DMAIC project and its impact on organizational performance. International Journal of Production Economics. 2015; 168: 105–117. https://doi.org/10.1016/j.ijpe.2015.06.007.
20. Lombardi R. Knowledge transfer and organizational performance and business process: past, present and future researches. Business Process Management Journal. 2019; 25: 2–9. https://doi.org/10.1108/BPMJ-02-2019-368.
21. Yunis M, Tarhini A, Kassar A. The role of ICT and innovation in enhancing organizational performance: The catalysing effect of corporate entrepreneurship. Journal of Business Research. 2018; 88: 344–356. https://doi.org/10.1016/j.jbusres.2017.12.030.
22. Ferlie E, Nicolini D, Ledger J, D'Andreta D, Kravcenko D, de Pury J. NHS top managers, knowledge exchange and leadership: the early development of Academic Health Science Networks – a mixed-methods study. Health Services Delivery Research. 2017; 5. https://doi.org/10.3310/hsdr05170.
23. Al Ahbabi SA, Singh SK, Balasubramanian S, Gaur SS. Employee perception of impact of knowledge management processes on public sector performance. Journal of Knowledge Management. 2019; 23: 351–373. https://doi.org/10.1108/JKM-08-2017-0348.
24. Oluikpe P. Developing a corporate knowledge management strategy. Journal of Knowledge Management. 2012; 16: 862–878. https://doi.org/10.1108/13673271211276164.
25. Grant RM. Towards a knowledge-based view of the firm. Strategic Management Journal. 1996; 17: 109–122. https://doi.org/10.1002/smj.4250171110.
26. Hislop D, Bosua R, Helms R. Knowledge management in organizations: A critical introduction. Oxford: Oxford University Press; 2018.

27. Kogut B, Zander U. Knowledge of the firm and the evolutionary theory of the multinational corporation. Journal of International Business Studies. 2003; 34: 516–529. https://doi.org/10.1057/palgrave.jibs.8400058.
28. Kogut B, Zander U. Knowledge of the Firm, Combinative Capabilities, and the Replication of Technology. Organization Science. 1992; 3: 383–397. https://doi.org/10.1287/orsc.3.3.383.
29. Barney J. Firm resources and sustained competitive advantage. Journal of Management. 1991; 17: 99–120. https://doi.org/10.1177/014920639101700108.
30. Martin SL, Javalgi RG. Explaining performance determinants: A knowledge based view of international new ventures. Journal of Business Research. 2019; 101: 615–626. https://doi.org/10.1016/j.jbusres.2019.02.041.
31. Inkinen H. Review of empirical research on knowledge management practices and firm performance. Journal of Knowledge Management. 2016; 20: 230–257. https://doi.org/10.1108/JKM-09-2015-0336.
32. Heathfield H, Louw G. New challenges for clinical informatics: knowledge management tools. Health Informatics Journal. 1999; 5: 67–73. https://doi.org/10.1177/146045829900500203.
33. Hopkins E. Knowledge Management in healthcare libraries: the current picture. Health Information and Libraries Journal. 2017; 34: 103–105. https://doi.org/10.1111/hir.12183.
34. Ali N, Tretiakov A, Whiddett D, Hunter I. Knowledge management systems success in healthcare: leadership matters. International Journal of Medical Informatics. 2017; 97: 331–340. https://doi.org/10.1016/j.ijmedinf.2016.11.004.
35. Cribb A. Improvement Science Meets Improvement Scholarship: Reframing Research for Better Healthcare. Health Care Analysis. 2018; 26: 109–123. https://doi.org/10.1007/s10728-017-0354-6.
36. Camargo-Borges C, Santos-Moscheta M. Health 2.0: Relational Resources for the Development of Quality in Healthcare. Health Care Analysis. 2016; 24: 338–348. https://doi.org/10.1007/s10728-014-0279-2.
37. Bloodgood JM, Salisbury WD. Understanding the influence of organizational change strategies on information technology and knowledge management strategies. Decision support systems. 2001; 31: 55–69. https://doi.org/10.1016/S0167-9236(00)00119-6.
38. Bohn RE. Measuring and managing technological knowledge. In: Neef D, GA S, Cefola J, editors. The Economic Impact of knowledge. Boston: Butterworth-Heinemann; 1998. pp. 295–314.
39. Choi B, Lee H. Knowledge management strategy and its link to knowledge creation process. Expert Systems with applications. 2002; 23: 173–187. https://doi.org/10.1016/S0957-4174(02)00038-6.
40. Fernandez N, Leduc N, Fon NC, Ste-Marie LG, Nguyen-Dinh D, Boucher A. Recognizing Change in Post-Graduate Medical Education Using the Organizational

Knowledge Creation Model. Creative Education. 2020; 11: 783–796. https://doi.org/10.4236/ce.2020.115056.

41. Lee H, Choi B. Knowledge management enablers, processes, and organizational performance: An integrative view and empirical examination. Journal of Management Information Systems. 2003; 20: 179–228. https://doi.org/10.1080/07421222.2003.11045756.
42. Zanetti CA, Taylor N. Value co-creation in healthcare through positive deviance. Healthcare. 2016; 4: 277–281. https://doi.org/10.1016/j.hjdsi.2016.06.003.
43. Kitson A, Brook A, Harvey G, Jordan Z, Marshall R, O'Shea R et al. Using complexity and network concepts to inform healthcare knowledge translation. International Journal of Health Policy and Management. 2018; 7: 231–243. https://doi.org/10.15171/ijhpm.2017.79.
44. Ayanbode OF, Nwagwu WE. Collaborative technologies and knowledge management in psychiatric hospitals in South West Nigeria. Information Development Epub ahead of print. 2020; 14. https://doi.org/10.1177/0266666919895563.
45. Vezzetti E, Moos S, Kretli S. A product lifecycle management methodology for supportive knowledge reuse in the consumer packaged goods domain. Computer-Aided Design. 2011; 43: 1902–1911. https://doi.org/10.1016/j.cad.2011.06.025.
46. Zhang X, Jiang JY. With whom shall I share my knowledge? A recipient perspective of knowledge sharing. Journal of Knowledge Management. 2015; 19: 277–295. https://doi.org/10.1108/JKM-05-2014-0184.
47. Scheegenannt Halfmann S, Evangelatos N, Kweyu E, DeVilliers C, Steinhausen K, van der Merwe A et al. The Creation and Management of Innovations in Healthcare and ICT. The European and African Experience Public Health Genomics. 2018; 21: 197–206. https://doi.org/10.1159/000499853.
48. Lo Presti L, Testa M, Marino V, Singer P. Engagement in healthcare systems: Adopting digital tools for a sustainable approach. Sustainability (Switzerland). 2019; 11: 1–15. https://doi.org/10.3390/su11010220.
49. Aceto G, Persico V, Pescapé A. The role of Information and Communication Technologies in healthcare: taxonomies, perspectives, and challenges. Journal of Network and Computer Applications Epub ahead of print. 2018. https://doi.org/10.1016/j.jnca.2018.02.008.
50. Argote L, Ingram P, Levine JM, Moreland RL. Knowledge Transfer in Organizations: Learning from the Experience of Others. Organizational Behavior and Human Decision Processes. 2000; 82: 1–8. https://doi.org/10.1006/obhd.2000.2883.
51. Prihodova L, Guerin S, Tunney C, Kernohan WG. Key components of knowledge transfer and exchange in health services research: Findings from a systematic scoping review. Journal of Advanced Nursing. 2019; 75: 313–326. https://doi.org/10.1111/jan.13836.

52. Coleman EA. Falling through the cracks: Challenges and opportunities for improving transitional care for persons with continuous complex care needs. Journal of the American Geriatrics Society. 2003; 51: 549–555. https://doi.org/10.1046/j.1532-5415.2003.51185.x.
53. Hastings SN, Heflin MT. A systematic review of interventions to improve outcomes for elders discharged from the emergency department. Academic Emergency Medicine. 2005; 12: 978–986. https://doi.org/10.1197/j.aem.2005.05.032.
54. Wenger NS, Young RT. Quality indicators for continuity and coordination of care in vulnerable elders. Journal of the American Geriatrics Society. 2007; 55: 285–292. https://doi.org/10.1111/j.1532-5415.2007.01334.x.
55. Aldohyan M, Al-Rawashdeh N, Sakr FM, Rahman S, Alfarhan AI, Salam M. The perceived effectiveness of MERS-CoV educational programs and knowledge transfer among primary healthcare workers: A cross-sectional survey. BMC Infectious Diseases. 2019; 19: 1–9. https://doi.org/10.1186/s12879-019-3898-2.
56. Radević I, Haček M. Decentralisation Processes in Montenegrin Public Administration: Challenges of Health System. Lex Localis – Journal of Local Self-Government. 2019; 17: 471–492. https://doi.org/10.4335/17.3.471-493(2019).
57. Wensing M, Grol R. Knowledge translation in health: How implementation science could contribute more. BMC Medicine. 2019; 17: 1–6. https://doi.org/10.1186/s12916-019-1322-9.
58. Jahn R, Müller O, Nöst S, Bozorgmehr K. Public-private knowledge transfer and access to medicines: A systematic review and qualitative study of perceptions and roles of scientists involved in HPV vaccine research. Globalization and Health. 2020; 16: 1–16. https://doi.org/10.1186/s12992-020-00552-9.
59. Hazlehurst C, Brouthers KD. IB and strategy research on “new” information and communication technologies: Guidance for future research. Progress in International Business Research. 2018; 13: 65–89. https://doi.org/10.1108/S1745-886220180000013004.
60. Canzano D, Grimaldi M. An integrated framework to implement a knowledge management programme: The role of technological tools and techniques. International Journal of Intelligent Enterprise. 2012; 1: 233–247. https://doi.org/10.1504/IJIE.2012.052554.
61. Liu Y, Lee Y, Chen ANK. Evaluating the effects of task–individual– technology fit in multi-DSS models context: A two-phase view. Decision Support Systems. 2011; 51: 688–700. https://doi.org/10.1016/j.dss.2011.03.009.
62. Vezzetti E. Study and development of morphological analysis guidelines for point cloud management: The “decisional cube.” Computer-Aided Design. 2011; 43: 1074–1088. https://doi.org/10.1016/j.cad.2011.01.002.
63. Nyame-Asiamah F. Improving the ‘manager-clinician’ collaboration for effective healthcare ICT and telemedicine adoption processes–a cohered emergent perspective.

Information Technology for Development. 2020; 26: 525–550. https://doi.org/10.1080/02681102.2019.1650326.

64. Tripathi VR, Popli M, Ghulyani S, Desai S, Gaur A. Knowledge creation practices at organizational boundaries: the role of ICT in sickle-cell care for tribal communities. Journal of Knowledge Management Epub ahead of print. 2020. https://doi.org/10.1108/JKM-09-2019-0521.

65. Wu MS. Information literacy, creativity and work performance. Information Development. 2019; 35: 676–687. https://doi.org/10.1177/0266666918781436.

66. Soto-Acosta P, Cegarra-Navarro JG. New ICTs for Knowledge Management in Organizations. Journal of Knowledge Management. 2016; 20: 417–422. https://doi.org/10.1108/JKM-02-2016-0057.

67. Ukachi NB, Anasi SNI. Information and communication technologies and access to maternal and child health information: Implications for sustainable development. Information Development. 2019; 35: 524–534. https://doi.org/10.1177/0266666918767482.

68. Papanastasiou G, Drigas A, Skianis C, Lytras M, Papanasstasiou E. Patient-centric ICTs based healthcare for students with learning, physical and/or sensory disabilities. Telematics and Informatics. 2018; 35: 654–664. https://doi.org/10.1016/j.tele.2017.09.002.

69. Sabeeh Z, Mustapha SMFD S, Mohamad R. Healthcare knowledge sharing among a community of specialized physicians. Cognition, Technology and Work. 2018; 20: 105–124. https://doi.org/10.1007/s10111-017-0453-z.

70. Dammaj A, Alawneh A, Abu Hammad A, Sweis RJ. Investigating the relationship between knowledge sharing and service quality in private hospitals in Jordan. International Journal of Productivity and Quality Management. 2016; 17: 437–455. https://doi.org/10.1504/IJPQM.2016.075248.

71. Fletcher-Brown J, Carter D, Pereira V, Chandwani R. Mobile technology to give a resource-based knowledge management advantage to community health nurses in an emerging economies context. Journal of Knowledge Management Epub ahead of print. 2020. https://doi.org/10.1108/JKM-01-2020-0018.

72. Krasniqi A, D'Huyvetter M, Devoogdt N, Frejd FY, Sörensen J, Orlova A et al. Same-Day Imaging Using Small Proteins: Clinical Experience and Translational Prospects in Oncology. Journal of Nuclear Medicine. 2018; 59: 885–891. https://doi.org/10.2967/jnumed.117.199901.

73. Harman HH. Modern factor analysis. Chicago: University of Chicago Press; 1976.

74. Podsakoff PM, Bommer WH, Podsakoff NP, MacKenzie SB. Relationships between leader reward and punishment behavior and subordinate attitudes, perceptions, and behaviors: A meta-analytic review of existing and new research. Organizational Behavior and Human Decision Processes. 2006; 99: 113–142. https://doi.org/10.1016/j.obhdp.2005.09.002.

75. Downes TV. An evaluation of knowledge management practices in nonprofit community services organisations in Australia. PhD Thesis, Southern Cross University. 2014.
76. Singh MD, Kant R. Knowledge management barriers: an interpretive structural modeling approach. International Journal of Management Science and Engineering Management. 2008; 3: 141–150. https://doi.org/10.1080/17509653.2008.10671042.
77. Debowski S. Knowledge Management. Wiley: Milton; 2006.
78. Anantatmula VS, Stankosky M. KM criteria for different types of organisations. International Journal of Knowledge and Learning. 2008; 4: 18–35. https://doi.org/10.1504/IJKL.2008.019735.
79. Riege A. Three-dozen knowledge-sharing barriers managers must consider. Journal of Knowledge Management. 2005; 9: 18–35. https://doi.org/10.1108/13673270510602746.
80. Bernerth JB, Aguinis H. A critical review and best- practice recommendations for control variable usage. Personnel Psychology. 2016; 69: 229–283. https://doi.org/10.1111/peps.12103.
81. Srivastava A, Bartol K, Locke E. Empowering Leadership in Management Teams: Effects on Knowledge Sharing, Efficacy, and Performance. The Academy of Management Journal. 2006; 49: 1239–1251. https://doi.org/10.5465/amj.2006.23478718.
82. Le PB, Lei H. How transformational leadership supports knowledge sharing: evidence from Chinese manufacturing and service firms. Chinese Management Studies. 2017; 11: 479–497. https://doi.org/10.1108/CMS-02-2017-0039.
83. Hair JF, Anderson RE, Tatham RL, Black WC. Multivariate Data Analysis. NJ: Prentice Hall; 1998.
84. Fornell C, Larcker DF. Evaluating structural equation models with unobservable variables and measurement error. Journal of Marketing Research. 1981; 18: 39–51. https://doi.org/10.2307/3151312.
85. Diamantopoulos A, Siguaw JA. Introducing LISREL. SAGE Publications Ltd; 2000. https://doi.org/10.4135/9781849209359.
86. Černe M, Jaklič M, Škerlavaj M. Management Innovation in Focus: The Role of Knowledge Exchange, Organizational Size, and IT System Development and Utilization. European Management Review. 2013; 10: 153–166. https://doi.org/10.1111/emre.12013.
87. Horvath H, Brindis CD, Reyes EM, Yamey G, Franck L, KTE Working Group. Preterm birth: The role of knowledge transfer and exchange. Health Research Policy and Systems. 2017; 15: 1–14. https://doi.org/10.1186/s12961-017-0238-0.
88. Alajmi BM, Marouf LN, Chaudhry AS. Knowledge Management for Healthcare: Investigating Practices that Drive Performance. Journal of Information and Knowledge Management. 2016; 15: 1–17. https://doi.org/10.1142/S0219649216500143.

Figure 1

Click here to access/download;Figure;Fig 1.tif

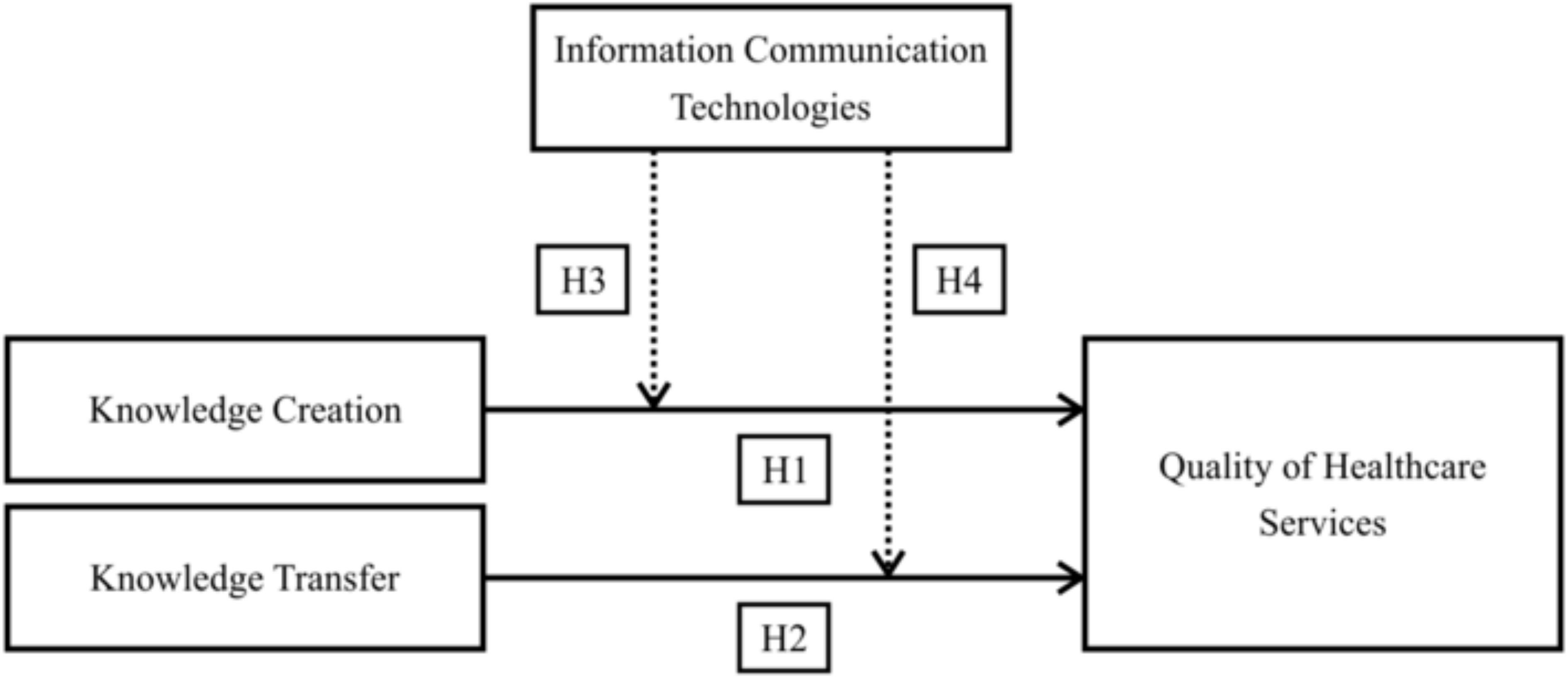

Figure 2

Click here to access/download;Figure;Fig 2.tif

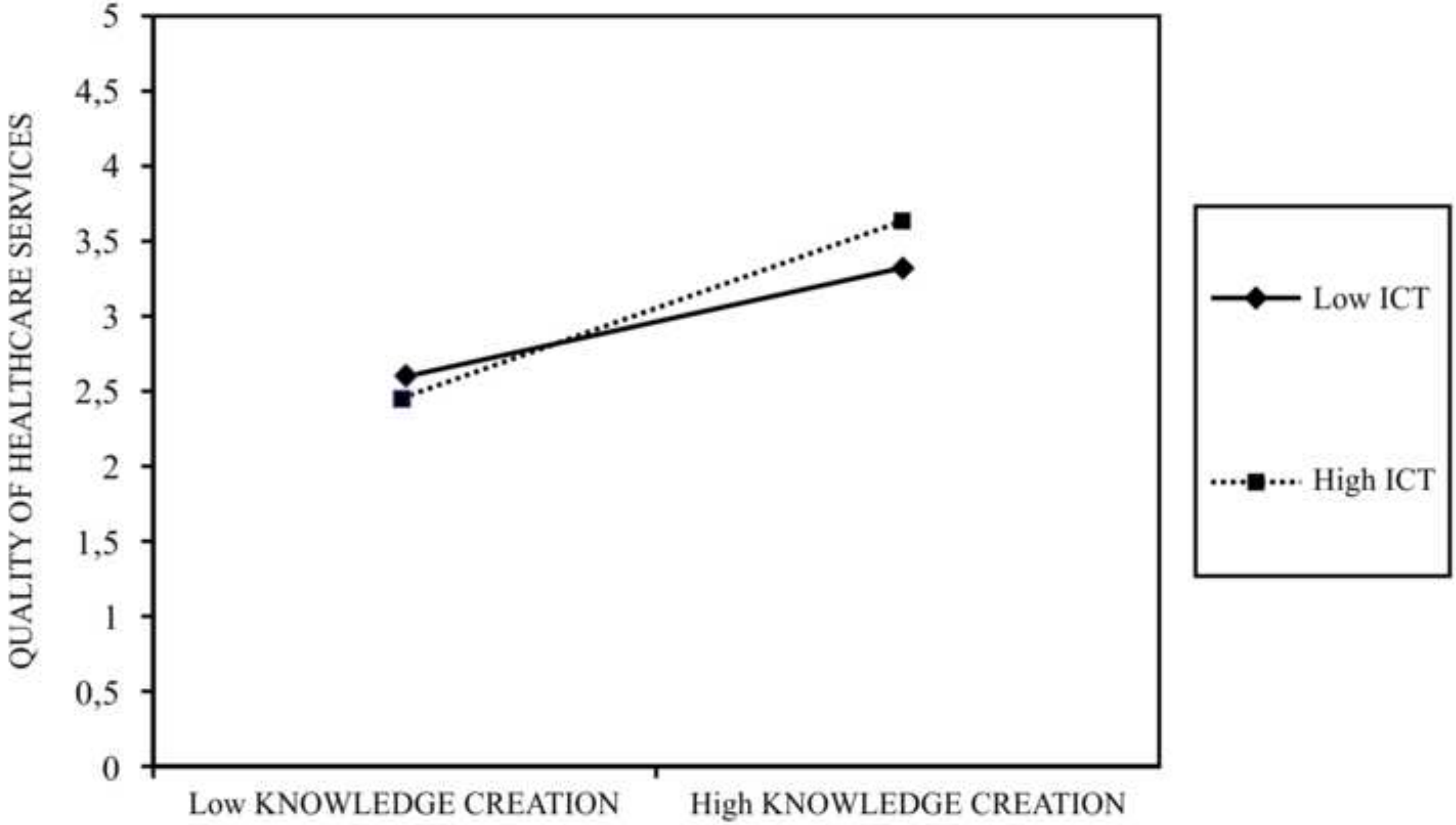

Figure 3

Click here to access/download;Figure;Fig 3.tif

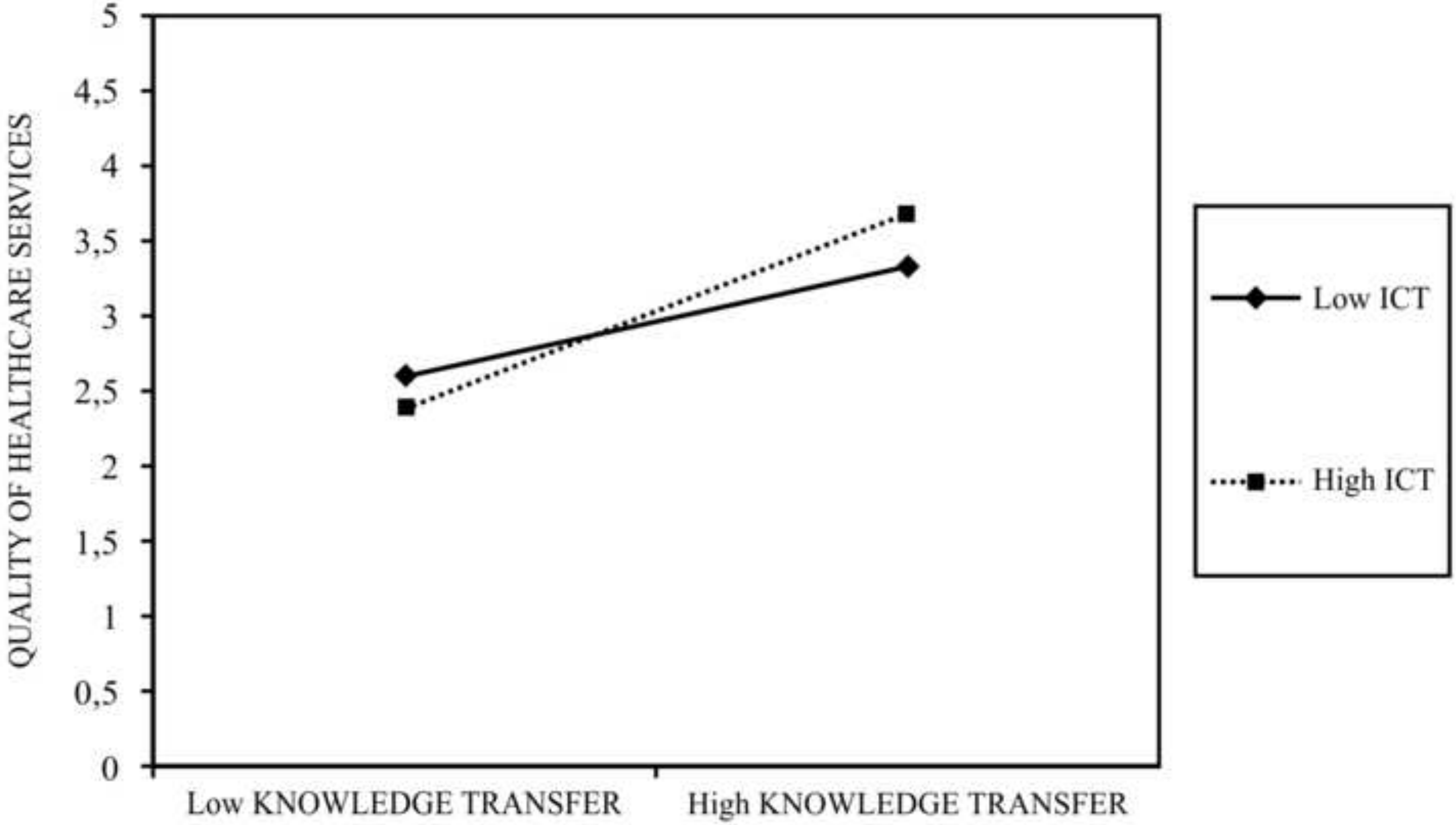

Click here to access/download
Supporting Information
S1 - DataShare.xlsx
