# Supplementary material for: The role of information communication technologies as a moderator of knowledge creation and knowledge sharing in improving the quality of healthcare services
Source: PLoS One. 2022 Aug 3;17(8):e0272346. doi: 10.1371/journal.pone.0272346 (PMC9348677; doi:10.1371/journal.pone.0272346)
Supplement: Attachment — Submitted filename: Response to Reviewers.docx [file pone.0272346.s003.docx]

**RESPONSE TO REVIEW COMMENTS**

Thank you for submitting your manuscript to PLOS ONE. After careful consideration, we feel that it has merit but does not fully meet PLOS ONE's publication criteria as it currently stands. Therefore, we invite you to submit a revised version of the manuscript that addresses the points raised during the review process.

Please submit your revised manuscript by Dec 19 2021 11:59PM. If you will need more time than this to complete your revisions, please reply to this message or contact the journal office at plosone@plos.org. When you're ready to submit your revision, log on to https://www.editorialmanager.com/pone/ and select the 'Submissions Needing Revision' folder to locate your manuscript file.

Please include the following items when submitting your revised manuscript:

- A rebuttal letter that responds to each point raised by the academic editor and reviewer(s). You should upload this letter as a separate file labeled 'Response to Reviewers'.
- A marked-up copy of your manuscript that highlights changes made to the original version. You should upload this as a separate file labeled 'Revised Manuscript with Track Changes'.
- An unmarked version of your revised paper without tracked changes. You should upload this as a separate file labeled 'Manuscript'.

If you would like to make changes to your financial disclosure, please include your updated statement in your cover letter. Guidelines for resubmitting your figure files are available below the reviewer comments at the end of this letter.

If applicable, we recommend that you deposit your laboratory protocols in protocols.io to enhance the reproducibility of your results. Protocols.io assigns your protocol its own identifier (DOI) so that it can be cited independently in the future. For instructions see: <https://journals.plos.org/plosone/s/submission-guidelines#loc-laboratory-protocols>. Additionally, PLOS ONE offers an option for publishing peer-reviewed Lab Protocol articles, which describe protocols hosted on protocols.io. Read more information on sharing protocols at <https://plos.org/protocols?utm_medium=editorial>-email&utm_source=authorletters&utm_campaign=protocols [1].

We look forward to receiving your revised manuscript.

Kind regards,

Nadja Damij, Ph.D.

Academic Editor,

PLOS ONE

**Answer to the editor:** Dear editor, we would like to thank you and the reviewers for your valuable feedbacks which helped us to improve our manuscript. We believe that the progress made in the manuscript is also heavily influenced by numerous valuable suggestions provided from our reviewers in the revision process. We hope you will find our responses satisfactory.

**Reviewer(s)' Comments to Author:**

**Reviewer: 1**

**1. A general comment about relevancy. We are all faced with the same issue, that after the pandemic nothing is the same any more, could you actualise on that?**

Answer to the review: Thanks for the comment. Definitely, the pandemic has changed and is currently changing the algorithms of business systems. Although in fact the data of our research were collected before the pandemic, we believe that they can be useful for the researchers, in order to repeat the same research and through a longitudinal approach enable a better understanding of the observed phenomena in the field of health. The pandemic showed how much the creation of knowledge is a condition for survival in a certain way (finding a vaccine). On the other hand, knowledge sharing has proven to be important, not only in a micro context - at the institution level, but also globally. Countries that later faced the challenges of the pandemic could benefit from the experiences of other countries and be more prepared to face the crisis. Inevitably, a pandemic affects business awareness. Crises are becoming a new normality, and the organization must create a system that will be fully adaptable and ready to put complete knowledge in the function of organizational goals. In that sense, the issues of knowledge creation, knowledge sharing, and the impact of technology on the quality of health services (growing participation of telemedicine) are increasingly important for the purpose of preserving health, ie for the purpose of increasing the quality of health services. Recognizing the fact that the challenges of dealing with the pandemic are still ongoing, in this paper, according to your esteemed comment, we have tried to actualize certain issues through the prism of the COVID-19 pandemic (in the Discussion chapter). We hope we have succeeded in achieving that goal.

**2. Some other research (**[**https://blogs.bmj.com/bmj/2019/05/01/cheng-hock-toh-current-geographical-spread-research-failing-patients/**](https://blogs.bmj.com/bmj/2019/05/01/cheng-hock-toh-current-geographical-spread-research-failing-patients/)**) have hypothesised, that there is a big discrepancy between centre and periphery. Might this be an explanatory factor also in your research?**

Answer to the review: Thank you for your comment. When exploring our data for only central organizations, we first explored the standardized factor loadings for all of our four constructs if they are statistically significant (>.50). One item intended to measure knowledge creation, one item intended to measure knowledge transfer, one item intended to measure quality of healthcare services and two items intended to measure information communication technologies did not meet the criteria in the literature and were therefore omitted from the “final” version of the central test model. Our “final” central test model consists of 15 items utilized to evaluate the existing state of four measured constructs. The model fit to data is as follows: CFI = 0.775 (original model in the proposed manuscript 0.97); chi-square 164.453 (original model in the proposed manuscript 104.690); RMSEA = .148 (original model in the proposed manuscript .07); and df = 74 (original model in the proposed manuscript 67). As CFI and RMSEA are below the recommended values in the literature, we concluded that it does not make sense to run any further tests on this specific central test model.

When exploring our data for only rural organizations, we again first explored the standardized factor loadings for all of our four constructs if they are statistically significant (>.50). Two items intended to measure quality of healthcare services and two items intended to measure information communication technologies did not meet the criteria in the literature and were therefore omitted from the “final” version of the rural test model. Our “final” rural test model consists of 16 items utilized to evaluate the existing state of four measured constructs. The model fit to data is as follows: CFI = 0.87 (original model in the proposed manuscript 0.97); chi-square 244.843 (original model in the proposed manuscript 104.690); RMSEA = .129 (original model in the proposed manuscript.07); and df = 104 original model in the proposed manuscript 67). As CFI and RMSEA are below the recommended values in the literature, we concluded that it does not make sense to run any further tests on this specific rural test model.

Therefore, we concluded that there might be some potential differences between central and rural healthcare organizations and their efforts to engage in knowledge management activities. However, we believe that our original model, combining both central and rural healthcare organizations better suits our research study and proposed constructs and adds more scientific value to the research community, especially when focusing on model fit to data.

**You will find my comments in the attached PDF.**

Answer to the review: Thanks for all the comments and suggestions you added in the PDF. We went through the comments very carefully, and took them into account in order to improve the quality of the text

Specifically:

1. "Not advisable that you use abbreviations in the title".

Thank you for your relevant and useful suggestion.

2. Can you tell something more about selection and recruitment? What was the sampling procedure used?

Yes of course. Thanks for the suggestion. We have enriched the manuscript with new details regarding the sample. We are also free to give you a more detailed explanation in this section. Thank you for your suggestion. As mentioned in our paper health institutions in Montenegro were selected as research units. In each health institution covered by our research, one member of the administration (usually the director), one medical doctor and one medical technician answered the questionnaire that measured the categories of organizational factors of knowledge management. Our approach was hopefully in line with the desired method of triangulation, intended to increase the validity of our results.

As per the Decision on the Network of Health Institutions, the network of public health institutions in Montenegro (founded by the state) consists of 18 health centres at primary level: Andrijevica, Bar, Berane, Bijelo Polje, Budva, Cetinje, Danilovgrad, Herceg Novi, Kolašin, Kotor, Mojkovac, Nikšič, Plav, Pljevlja, Podgorica, Rožaje, Tivat and Ulcinj (Article 3). The Institute of Public Health carriers out primary health care services too (even though it is a public health institution at the tertiary level with associated activities - Article 50 of the Law on Health Care). Eight general hospitals provide health care services (Bar, Berane, Bijelo Polje, Cetinje, Kotor, Meljine, Nikšić, Pljevlja) and three specialized hospitals (Brezovik, Dobrota, Risan). The Clinical Centre of Montenegro provides hospital health care services at the secondary and tertiary level, as well as two private health institutions - Hospital "Codra" and Polyclinic "Tesla Medica" Berane (Article 10). The Emergency Centre of Montenegro provides emergency medical care at the primary level of health care. The Institute for Blood Transfusion of Montenegro is responsible for blood transfusion (Articles 12-13). In addition, the Institute of Public Health of Montenegro is a part of the network of health institutions in Montenegro that provides services at the primary, secondary and tertiary level.

Therefore, out of a total of 35 health care institutions that make the network of health care institutions in Montenegro, our sample included 34 (Meljine Hospital, although formally speaking it belongs to the network of health care institutions did not operate at the time of research (it was in the closing stage of its operations) hence it was not included in the sample). In addition to 34 health care institutions, of which 32 are public and 2 large-size private institutions, the sample included 11 small and medium-sized private health care institutions, to make sure the sample of private health care institutions is more representative, by including smaller and larger health care institutions, as well as institutions from all three regions of the country (centre, north and south). In addition, the number of respondents from public and private health care institutions corresponds to the market share of both institutions (more than 2/3 of health care services in Montenegro are provided through public health care institutions). Please note that the sample excluded pharmacies and institutions that work in delivery of medicines and medical devices respectively.

The research was therefore conducted on a sample of the following 45 health institutions:

1. Health centres (18 institutions) in: Andrijevica, Bar, Berane, Bijelo Polje, Budva, Cetinje, Danilovgrad, Herceg Novi, Kolašin, Kotor, Mojkovac, Nikšić, Plav, Pljevlja, Podgorica, Rožaje, Tivat and Ulcinj;
2. General hospitals (seven institutions) in: Bar, Berane, Bijelo Polje, Cetinje, Kotor, Nikšić and Pljevlja; Note: Meljine Hospital.
3. Special hospitals (three institutions) in: Brezovik, Dobrota and Risan;
4. Institute of Public Health in Podgorica, Clinical Centre of Montenegro in Podgorica, Emergency Centre in Podgorica, and Institute of Physical Medicine, Rehabilitation and Rheumatology "Dr Simo Milošević" AD Igalo;
5. Private health institutions (13 institutions): Biochemical and microbiological laboratory "In Vitro" in Podgorica, Codra Hospital in Podgorica, Krapović Medical in Budva, Milmedika Nikšić, Eye clinic "Borović" in Podgorica, Eye clinic "Dr Lutovac" in Berane , Polyclinic Hippocrates in Podgorica, Polyclinic Merkur Nera in Podgorica, Spa Medica Podgorica, Specialist hospital of internal medicine "Dr Masonicic" in Bar, Special hospital "Dr MedTim" in Podgorica, Dental practice "Musura" in Budva, Tesla Medical in Berane.

In conclusion, our final sample included both public (32) and private (13) health care institutions. In addition, the sample encompassed institutions that provide health services at the primary, secondary and tertiary levels. Lastly, the sample included health care institutions from all three regions of the country (centre, north and south). In addition to 45 health care institutions, from which 3 respondents each (135 respondents) were interviewed, the sample included 16 members of the Union of Medical Doctors. This was done in order to improve objectivity of evaluations and representativeness of the sample. With the view to the above and given the complexity of the organization of health system and relatively limited availability of respondents, we hope that the final sample of 151 respondents meets the criterion of representativeness.

From a theoretical perspective, collecting data from employees from public health institutions and from private health institutions was conducted utilizing a combination of a convenient and snowball sample. The convenient sample can be understood within the category of non-random samples that implies the selection of members of the population who are available at a specific time. A snowball sample is a type of non-random sampling, in which respondents help the researcher in identifying other respondents and help establishing cooperation between them. Although, random sampling strategies can be considered the gold standard, in practice, random sampling of participants is not always feasible nor necessarily the optimal choice (van Hoeven et al., 2015), as was the case in our research. When choosing a sample of participants, researchers are often faced with a trade-off between the wish for randomization and pragmatic considerations. Typically, random sampling is considered as the gold standard of sampling strategies due to its unbiasedness and the possibility to evaluate the reliability (precision) of the resulting estimates (Banerjee & Chaudhury, 2010; Levy & Lemeshow, 2008; Tiwari & Chilwal, 2014). However, as previously mentioned, random sampling is not always possible and / or feasible in practice, in particular due to constraints in time, resources and costs, and researchers in the medical field often use a “convenience” or a purposive sample, i.e. by choosing participants who are easy to recruit. In addition, studies exist that claim that such purposive strategies can lead to representative samples (Raaijmakers et al., 2008; Topp et al., 2004), where a sample is considered representative when either sample characteristics or inferences from the sample approximate population values. Moreover, the statement that a random sample is unbiased posits that it will provide a representative sample on average. The probability of randomly drawing an unrepresentative sample is large if your population is small (van Hoeven et al., 2015) (not the case in our research).

We include some references that are related to the health care system in Montenegro:

1. Decision on the network of health institutions (Official Gazette of Montenegro No. 037/16 as of 16 June 2016).
2. Decision on amendments to the decision on the network of health institutions (Official Gazette of Montenegro No. 048/17 as of 24 July 2017).
3. Decision on amendments to the decision on the network of health institutions (Official Gazette of Montenegro No. 012/18 as of 23 February 2018); day of entry into force is indicated in each decision. <https://fzocg.me/Administracija/Documents/1570176764Odluka%20o%20mre%c5%bei%20zdravstvenih%20ustanova.pdf>
4. “not needed”

You're right. Thanks for this comment. We agree. We have made corrections within the text.

5. Do you have any information where are these units located, are they all central? <https://blogs.bmj.com/bmj/2019/05/01/cheng-hock-toh-current-geographical-spread-research-failing-patients/>

Thank you for your question. We have the data. You can find the answer in the previous part of the text. Montenegro is a territorially small country, with 13,888 km2 and 622,028 inhabitants. However, from the aspect of geographical dispersion of the observed units of analysis, the sample includes respondents from all three regions (northern, central and southern). Just before the description of the sample, we added information on how many institutions are from which region.

6. Methods section might start from here

Thanks for the suggestion. You're right. We agree and we have necessary corrections in the manuscript.

7. Maybe be specific (common method bias)

Thanks, you're right. We tried to be more specific, and changed the content of a specific sentence. We hope it is acceptable now.

8. Maybe you can provide citation for the measurement instruments used?

You're right. We apologize for missing this in the first version. As you can see, we have referenced 5 scientific papers in which researchers have used the same measuring instruments as we have used in this paper. If you think that this part needs to be further strengthened, we are ready to do so.

9. move this to the results section

We have moved this sentence to the beginning of the results section, which you can see in the manuscript. Thanks for the helpful suggestion and remarkable observation.

10. Teaching hospitals - since they have the interest to be involved in research?

Yes, thanks for this correction, which will improve the quality of our manuscript in one segment. You can also find this change in the corrected version of the manuscript.

11. The fact that the data were collected before the pandemic might me seen as a positive thing in sense of preempting and providing preparedness. On the other hand, during the pandemic a ICT revolution happened, so many technical solution were developed almost over night, the development was fast and smooth, what was not possible before became a reality as soon as the fist lock down started. My suggestion is that you put a bit of thought in explaining which element of your research do you think would have changed if you would repeat the survey today?

Many thanks for your suggestion. We have discussed this topic in the paperwork. You can find our amendments via track changes.

12. How (to promote)?

We will make the results of the research available to the general public through the organization of seminars at higher education institutions in Montenegro with representatives of health authorities at the state and local levels. This is also against the background of the current drafting of the Public Administration Reform Strategy, which aims to enable greater accessibility and quality of health services provided to citizens through the process of digitalization, among others. In addition, the results of the research will be available to all managers of public and private clinics in Montenegro who participated in the research, to which we committed in the data collection process, with special reference to the cross-section of the current situation, detected shortcomings and ways ICT technology can have positive implications on the process of knowledge creation and dissemination.

13. I would also add either as a limitation or future research, that a mixed-method approach is needed to approach such a complex area and a complex setting. Qualitative research would add an in-depth explanation to it.

Thanks for this suggestion. We fully agree with this statement.

Mixed research methods offer an option that actually tries to take advantage of the similarities and differences in qualitative and quantitative methods. It represents a pragmatic alternative — showing how research can proceed without resolving the potential conflicts in worldviews. As a result, contemporary supporters of mixed research methods have made an intense effort to define, document, and classify it (Yin, 2011). This method is increasingly present in health research. In addition to health, it is often used in the fields of education, law, business, medicine, social work, etc. (Radević, 2021).

***Answer to the reviewer:*** *We would like to thank you for your precise revision. We have improved part of the text according to your valuable suggestions. Your review enabled us to solve many important issues that needed to be addressed within the scope of our paper. Your clear instructions were of paramount importance to improve our paper, with our intention to fully follow your recommendations. Thank you for your effort and precise revision.*

**Reviewer(s)' Comments to Author:**

**Reviewer: 2**

**1. The paper addresses an interesting and relevant topic. Overall, the paper is well-organised with good readability.**

Answer to the review: Thank you for your positive encouragement. The manuscript was significantly revised according to your valuable comments and suggestions.

**2. However, the contributions are unclear in terms of originality and implications to theory and practice within the knowledge management discipline. The title of the paper suggests an enabling role of ICT for knowledge management. Yet, a rich discussion on the role of ICT infrastructure (platforms, tools, etcetera), digital skills and governance in fostering knowledge creation and sharing is missing.**

Answer to the review: Thank you for your important comment. We totally agree and thank you again for your positive encouragement to define a more precise statement of the theoretical and practical importance of our work. Therefore, the following text to better emphasize our implications to theory has been added: With our research, we are able to add to previous theoretical findings within the context of the knowledge management discipline that posit that individual knowledge management activities are typically in a positive relationship with organizational performance (McIver and Lepisto, 2017). In a similar vein, we add to contemporary knowledge management theory that recognizes knowledge as a critical resource for the functioning of organizations (Willem and Buelens, 2007), including healthcare organizations as their performance is being inextricably tied to efficient use of knowledge (Richards and Duxbury, 2015).

Moreover, our research offers theoretical guidance to healthcare employees that rely on information-communication technologies to cope with a continuously increasing need to manage knowledge (Ali et al., 2012) and extends the research on success determinants of knowledge management within the healthcare setting. As such, our research is one of the few studies that explore the effects of organizational factors on knowledge management initiatives in healthcare organizations as suggested by Ali et al. (2017). Similarly, our study is following the proposition of Siong et al. (2011) that the role of knowledge and knowledge management is attracting increased attention from scholars and practitioners as an effort to achieve organizational excellence.

Attached please find the references newly included in the revised manuscript for better clarity of our explanations:

1. Ali N, Tretiakov A, Whiddett D, et al. (2017) Knowledge management systems success in healthcare: leadership matters. International Journal of Medical Informatics 97: 331-340. DOI: 10.1016/j.ijmedinf.2016.11.004.
2. Ali N, Whiddett D, Tretiakov A, et al. (2012) The use of information technologies for knowledge sharing by secondary healthcare organisations in New Zealand. Int. J. Med. Inform. 81(7): 500-506.
3. McIver D. and Lepisto DA (2017) Effects of knowledge management on unit performance: examining the moderating role of tacitness and learnability. Journal of Knowledge Management 21(4): 796-816. DOI: 10.1108/JKM-08-2016-0347.
4. Richards GS and Duxbury L (2015) Work-group Knowledge Acquisition in Knowledge Intensive Public-Sector Organization: An Exploratory Study. Journal of Public Administration Research and Theory 25(4): 1247-1277. DOI: 10.1093/jopart/muu034.
5. Siong CC, Salleh K, Syed Noh Syed A and Syed-Ikhsan SOS (2011) KM implementation in a public sector accounting organization: an empirical investigation. Journal of Knowledge Management 15(3): 497-512. DOI: 10.1108/13673271111137457.
6. Willem A and Buelens M (2007) Knowledge sharing in public sector organizations: the effect of organizational characteristics on interdepartmental knowledge sharing. Journal of Public Administration Research and Theory 17(4): 581-606. DOI: 10.1093/jopart/mul021.

To provide further insight into practical implications of our research, the following text has been added: *In line with McIver and Lepisto (2017), our study provides further insight into the knowledge management discipline aspect that is focused on findings that enable organizations to achieve competitive advantage in practice by managing and utilizing what they know or even what they will need to know. Such a state is particularly useful in the contemporary knowledge intensive economy. Another learning outcome for practitioners might be that knowledge-based organizations should not only blindly apply knowledge management related initiatives as they need to align them with activities on how to motivate, support and offer relevant knowledge to individual employees for knowledge initiatives to be successful. In such a situation, the role of managers is integral as they need to motivate their employees to be engaged and utilize available resources to improve their individual and organizational performance (Butt et al., 2019). As healthcare can be considered a practice-based profession, the goal of knowledge management in practice would be aimed to add value to services and to increase social wellbeing, societal effectiveness and general welfare (Myers, 2014; Ortenblad & Koris, 2014).*

Attached please find the references newly included in the revised manuscript for better clarity of our explanations:

1. Butt MA, Nawaz F, Hussain S, et al. (2019) Individual knowledge management engagement, knowledge-worker productivity, and innovation performance in knowledge-based organizations: the implications for knowledge processes and knowledge-based systems. Computational and Mathematical Organization Theory 25: 336-356. DOI: 10.1007/s10588-018-9270-z.
2. McIver D. and Lepisto DA (2017) Effects of knowledge management on unit performance: examining the moderating role of tacitness and learnability. Journal of Knowledge Management 21(4): 796-816. DOI: 10.1108/JKM-08-2016-0347.
3. Myers PS (2014) A Normative Model of Knowledge Management Effectiveness. In A Ortenblad (Ed.) Handbook of Research on Knowledge Management: Adaptation and Context (28-52). Cheltenham and Northampton, UK: Edward Elgar.
4. Ortenblad A and Koris R (2014) Is the Learning Organization Idea Relevant to Higher Education Institutions? A Literature Review and a Multi-Stakeholder Contingency Approach. International Journal of Educational Management 28(2): 173-214. DOI: doi.org/10.1108/IJEM-01-2013-0010.

In our effort to add to a more clear discussion on the role of ICT, the following text has been added: *Information-communication technology systems with their support to knowledge management processes positively influence the competitiveness of organizations (Andreeva and Kianto, 2012). As internet technology enables rapid search, access, exchange and retrieval of information it is deemed as suitable for collaboration and knowledge exchange between organizational members (Lucio-Nieto et al., 2012). Moreover, such systems typically support knowledge management practices as they facilitate knowledge acquisition and creation, knowledge dissemination, knowledge conversion and knowledge utilization (Martelo-Landroguez and Cegarra-Navarro, 2014; Palacios-Marques et al., 2015). Typically, technology is viewed an essential component and integral facilitator in any knowledge management initiative (Olobunmi Omotayo, 2015). With appropriate training and education for employees, such solutions have become crucial to organizations as they carry out many tasks related to knowledge management (Soto-Acosta and Cegarra-Navarro, 2016).*

Attached please find the references newly included in the revised manuscript for better clarity of our explanations:

1. Andreeva T and Kianto A (2012) Does knowledge management really matter? Linking knowledge management practices, competitiveness and economic performance. Journal of knowledge management 16(4): 617–636. DOI: 10.1108/13673271211246185.
2. Lucio-Nieto T, Colomo-Palacios R, Soto-Acosta P, Popa S and Amescua-Seco A (2012) Implementing an IT service information management framework: the case of COTEMAR, Int. J. Inf. Manage. 32(6): 589-594. DOI: 10.1016/j.ijinfomgt.2012.08.004.
3. Martelo-Landroguez S and Cegarra-Navarro JG (2014) Linking knowledge corridors to customer value through knowledge process. Journal of Knowledge Management 18(2): 342:365. DOI: 10.1108/JKM-07-2013-0284.
4. Olobunmi Omotayo F (2015) Knowledge Management as an Important tool in Organisational Management: A Review of Literature. Library Philosophy and Practice, Spring 4, 23 pages.
5. Palacios-Marques D, Soto-Acosta P and Merigo JM (2015) Analyzing the effects of technological, organizational and competition factors on Web knowledge exchange in SMEs. Telematics and Informatics 32(1): 23-32. DOI: 10.1016/j.tele.2014.08.003.
6. Soto-Acosta P and Cegarra-Navarro JG (2016) New ICTs for Knowledge Management in Organizations. Journal of Knowledge Management 20(3): 417–422. DOI: 10.1108/JKM-02-2016-0057.

Moreover, to better clarify the relationship between ICT and knowledge creation, the following text has been added: *Previous research of Lopez-Nicolas and Soto-Acosta (2010) supports a significant positive impact of having an appropriate information-communication technology infrastructure on knowledge creation. In a similar vein, Sambamurthy and Subramani (2005) have highlighted the critical role of information-communication technologies in shaping organizational efforts for knowledge creation. In addition, researchers Davenport and Prusak (1998) and Roberts (2000) posit that information-communication technology is a crucial aspect of knowledge creation due to the fact that such technologies facilitate speedy collection, storage and exchange of knowledge. While knowledge creation can be conducted without the support of information-communication technologies, such technology allows knowledge creation to be performed in a more effective manner (Robey et al., 2002). As state-of-the-art research supports the fact that information-communication technologies are an important enabler of the knowledge creation process, such technology must be designed an utilized in a manner that it is aligned with other organizational resources, with a particular emphasis on human resources, namely employees (Nonaka and Nishiguchi, 2001).*

Attached please find the references newly included in the revised manuscript for better clarity of our explanations:

1. Davenport TH and Prusak L (1998) Working knowledge: How organizations manage what they know. Boston, MA: Harvard Business School Press.
2. Lopez-Nicolas C and Soto-Acosta P (2010) Analyzing ICT adoption and use effects on knowledge creation: An empirical investigation in SMEs. International Journal of Information Management 30 521-528. DOI: 10.1016/j.ijinfomgt.2010.03.004Get.
3. Nonaka I and Nishiguchu T (2001). Knowledge emergence. New York: Oxford University Press.
4. Roberts J (2000) From know-how to show-how? Questioning the role of information and communication technologies in knowledge transfer. Technology Analysis & Strategic Management 12(4): 429-443. DOI: 10.1080/713698499.
5. Robey D, Ross JW and Boudreau MC (2002). Learning to implement enterprise systems: An exploratory study of the dialectics of change. Journal of Management Information Systems 19(1): 17-46. DOI: 10.1080/07421222.2002.11045713.
6. Sambamurthy V and Subramani M (2005) Knowledge problems in organizations: Foreword to special issue on information technologies and knowledge management. MIS Quarterly, 29(1): 1-7.

To improve our manuscript covering the part of ICT in relation to knowledge sharing, the following text has been added: *In previous research, information-communication technology was identified as an organizational factor that influences knowledge sharing at individual and team level (Farooq, 2018). In contemporary research, information-communication technology has been proposed as one of the crucial enablers of knowledge sharing (Panahi et al., 2016). Information-communication technology can also have a potentially significant influence on the knowledge sharing activity (Hendriks, 1999) as it provides the infrastructure that enables the establishment, maintenance, and intensification of relationships within and among teams (Huysman and Wulf, 2004). Contemporary information-communication technology systems that include also social networks can help employees to share their knowledge through common platforms and enable electronic storage of information and knowledge. Furthermore, information-communication technology systems can facilitate collaboration between employees and teams, and enrich their communication through various modern tools (Olobunmi Omotayo, 2015). Moreover, information-communication technology can accelerate access to information and knowledge that is stored in databases to enhance the knowledge sharing process in organizations. Such technology can support knowledge sharing by enabling effective communication channels and tools and by identifying the source of information or knowledge (Farooq, 2018). In a similar vein, Riege (2005) is of the opinion that information-communication technology enables instant access to large amounts of information and knowledge to facilitate long-distance collaboration and knowledge sharing between employees and organizations.*

Attached please find the references newly included in the revised manuscript for better clarity of our explanations:

1. Farooq R (2018) A conceptual model of knowledge sharing. International Journal of Innovation Science 10(2): 238-260. DOI: 10.1108/IJIS-09-2017-0087.
2. Hendriks P (1999) Why share knowledge? The influence of ICT on the motivation for knowledge sharing. Knowledge and Process Management 6(2): 91-100. DOI: 10.1002/(SICI)1099-1441(199906)6:2<91::AID-KPM54>3.0.CO;2-M.
3. Huysman M and Wulf V (2004) Social capital and information technology: current debates and research. In M Huysman and V Wulf (Eds.), Social capital and information technology (1-16). Cambridge, Massachussets, USA: MIT Press.
4. Olobunmi Omotayo F (2015) Knowledge Management as an Important tool in Organisational Management: A Review of Literature. Library Philosophy and Practice, Spring 4, 23 pages.
5. Panahi S, Watson J and Partridge H (2016) Conceptualising social media support for tacit knowledge sharing: physicians’ perspectives and experiences. Journal of Knowledge Management 20(2): 344-363. DOI: 10.1108/JKM-06-2015-0229.
6. Riege A (2005) Three-dozen knowledge-sharing barriers managers must consider. Journal of Knowledge Management 9(3): 18-35. DOI: 10.1108/13673270510602746.

**3. The literature review does not reveal the gaps in existing research
that justify the paper's originality.**

Answer to the review: Thank you for your relevant suggestion. To improve this part of our manuscript, we try to enhance the gap in existing research exploiting knowledge creation and quality of healthcare services. The following text has been added: *This study extends previous research that argues that the creation of knowledge is a crucial aspect of providing quality services in healthcare practice (El Morr and Subercaze, 2010). In a similar vein, our study extends existing research on knowledge creation as the final outcome of the process that enhances the quality and quantity of healthcare organization’s knowledge base (Yang et al., 2017), which subsequently influences the quality of services that are provided in practice to users. Moreover, our research answers the calls of research to provide suggestions on how can healthcare organizations can effectively deal with complex challenges such as knowledge creation for the successful and quality functioning of the healthcare system as a whole (Yang et al., 2017).*

Attached please find the references newly included in the revised manuscript for better clarity of our explanations:

1. El Morr C and Subercaze J (2010) Knowledge management in healthcare. In: Handbook of Research on Development in E-Health and Telemedicine: Technological and Social Perspectives. London: IGI Global.
2. Yang CW, Fang SC, Yan YH and Huang CY (2017) Gaining Legitimacy, Knowledge Creation and Hospital Survival. Journal of Public Health Issues and Practices Volume 1: 102. DOI: 10.33790/jphip1100102.

Similarly, to provide additional insight into the necessity to further research on the topic of knowledge sharing and quality of healthcare services, the following text has been added to clarify some of the gaps found in existing research: *With our study, we add to previous research that argues that healthcare teams frequently consist of interdisciplinary members that need to transfer knowledge to one another to be able to increase existing knowledge and create new knowledge and adequate healthcare solutions, which in practice improves the quality of services that are provided (Kessel et al., 2012). Historically, knowledge transfer in the healthcare environment was hindered due to numerous reasons. In the future, academics and practitioners are aiming to find improvements in knowledge transfer activities among healthcare professional as it can represent a tool to ultimately improve the quality of healthcare services (Ting et al., 2011).*

Attached please find the references newly included in the revised manuscript for better clarity of our explanations:

1. Kessel M, Kratzer J and Schultz C (2012) Psychological Safety, Knowledge Sharing, and Creative Performance in Healthcare Teams. Creativity and innovation management 21(2): 147-157. DOI: 10.1111/j.1467-8691.2012.00635.x.
2. Ting SL, Wang WM, Tse YK and Ip WH (2011) Knowledge elicitation approach in enhancing tacit knowledge sharing. Industrial Management & Data Systems 111(7): 1039-1064. DOI: 10.1108/02635571111161280.

According to your valuable insight, we provide novel findings that enhance the gap found in existing literature related to ICT and quality of healthcare services. Therefore, the following text has been added: *Our study adds to existing state-of-the-art literature that provides insight into the fast growing field of information-communication technologies that support the successful functioning of the healthcare sector with the provision of safe, efficient, high-quality, and information-communication technology assisted healthcare services. The provision of such services is typically reliant on an adequate workforce, financial resources and the knowledge and know-how on how to utilize services (Alpay et al., 2009). Moreover, our research builds the body of literature that promotes the positive impact of information-communication technology adoption on the safety, efficiency and quality of services in healthcare organizations (Agarwal and Ganesh, 2017) within the specific context of the Montenegro healthcare environment.*

*With our paper, we build on the notion that technological factors as for example information-communication infrastructure, technological skills, knowledge management tools and availability of technological facilities partially define the success of such interventions within the healthcare environment. Moreover, such technological factors are becoming more and more important in the successful provision of high-quality healthcare services in practice (Pankomera and van Greunen, 2020). Moreover, with our research we aim to close the gap of understanding on how information-communication technology can be used to promote the quality of services provided for patients (Groves et al., 2016). In a similar vein, we add to the body of literature that emphasizes the connection between information-communication technology and quality of healthcare services, which was a field that was partially neglected in the past due to an emphasis on technological interventions that offer mainly financial benefits (Clauser et al., 2011).*

Attached please find the references newly included in the revised manuscript for better clarity of our explanations:

1. Agarwal V and Ganesh L (2017) Implementing Quality Healthcare Strategies for Improving Service Delivery at Private Hospitals in India. Journal of Health Management 19(1): 159-169. DOI: 10.1177/0972063416682638.
2. Alpay L, Verhoef J, Xie B, Te’eni D and Zwetsloot-Schonk JH (2009) Current challenge in consumer health informatics: Bridging the gap between access to information and information understanding. Biomedical Informatics Insights 2(1): 1-10. DOI: 10.4137/BII.S2223.
3. Clauser SB, Wagner EH, Bowles EJA, Tuzzio L and Greene SM (2011) Improving modern cancer care through information technology. Am J Prev Med. 40(5): 198-207. DOI: 10.1016/j.amepre.2011.01.014.
4. Groves P, Kayyali B, Knott D and Kuiken SV (2016) The'big data'revolution in healthcare: accelerating value and innovation. 2016.
5. Pankomera R and van Greunen D (2020) ICT framework to support a patient-centric approach in public healthcare: A case study of Malawi. The Journal of Community Informatics 16: 45-76.

**4. The complexity of the knowledge creation/sharing issues in healthcare
services, which are typically led by evidence-based knowledge and
practices by clinicians/experts, is missing in the literature review.**

Answer to the review: You are right. According to your meticulous review we have added the following text to explain the complexity of knowledge creation in healthcare, with a particular emphasis on the evidence-based approach: *Knowledge creation is an important aspect of development and implementation of high-quality services and products (Orlikowski, 2002). As such it is perhaps even the most crucial aspect in the complex environment of healthcare, where evidence typically gained from empirical research supports the efficiency of utilizing limited available resources (Greenhalgh et al., 2004). Moreover, knowledge creation is of the primary activities of knowledge intensive organizations (Bettencourt et al., 2002), including healthcare organizations. Healthcare organizations heavily rely on clinical knowledge for delivering services in practice (Wu and Hu, 2012). Therefore, healthcare employees who create and transfer clinical knowledge have a paramount role in knowledge management activities in healthcare organizations (Davenport, 2005; Sivagiri, 2019). In addition, appropriately implementing knowledge creation in practice is especially complicated in the healthcare environment that is often characterized by evidence-based practice, where making decisions on how to provide or improve healthcare is related to integrating best available research evidence with a combination of clinical expertise and patient values, knowledge and preferences (Sackett et al., 1996).*

Attached please find the references newly included in the revised manuscript for better clarity of our explanations:

1. Bettencourt LA, Ostrom AL, Brown SW and Roundtree RI (2002) Client co-production in knowledge-intensive business services. California Management Review 44(4): 100-128.
2. Davenport TH (2005) Thinking For a Living: How to Get Better Performance and Results from Knowledge Workers. Harvard Business Press, Boston, MA.
3. Greenhalgh T, Robert G, Macfarlane F, Bate P and Kyriakidou O (2004) “Diffusion of innovations in service organizations: systematic review and recommendations.” The Milbank Quarterly 82(12): 581-629. DOI: 10.1111/j.0887-378X.2004.00325.x.
4. Orlikowski WJ (2002). “Knowing in practice: enacting a collective capability in distributed organizing.” Organization Science 13(3): 249-273. DOI: 10.1287/orsc.13.3.249.2776.
5. Sackett DL, Rosenberg WM, Gray JM, Haynes RB and Richardson WS (1996) Evidence based medicine: what it is and what it isn't. Br Med J. 312: 71. DOI: 10.1136/bmj.312.7023.71.
6. Sivagiri N (2019) The effects of knowledge management process on knowledge management effectiveness of physicians: an empirical study in multi-speciality hospitals. Int. J. Manag.
7. Wu IL and Hu YP (2012). Examining knowledge management enabled performance for hospital professionals: a dynamic capability view and the mediating role of process capability. J. Assoc. Inf. Syst. 13(12): 976-999. DOI: 10.17705/1jais.00319.

In a similar vein, to add to our manuscript the missing part of the complexity of knowledge sharing in healthcare, with a special focus on the evidence-based approach, the following text has been added: *In such a state, the contemporary and relevant knowledge has to be effectively management and shared among healthcare employees to improve the quality of services. Therefore, efficient knowledge sharing activities are crucial for healthcare organizations (Wu et al., 2021). Authors Shahmoradi et al. (2017) highlight that knowledge sharing plays a particularly important role in knowledge management activities within the evidence-based practice that is common within the healthcare setting. As healthcare organizations have a direct impact on people’s quality of life and wellbeing, the effectiveness of knowledge sharing is even more important in organizations that function in such a delicate environment (Sabeeh et al., 2018). Crass and Peters (2014) further highlight the complexity of knowledge in healthcare as they posit that the majority of innovations and the delivery of services are reliant on the skills and know-how of employees in healthcare organizations.*

Attached please find the references newly included in the revised manuscript for better clarity of our explanations:

1. Crass D and Peters B (2014) Intangible assets and firm-level productivity. ZEW-Cent Eur Econ Res: 14-120. DOI: 10.2139/ssrn.2562302.
2. Sabeeh Z, Syed Mustapha S and Mohamad R (2018) Healthcare knowledge sharing among a community of specialized physicians. Cognition, Technology and Work 20(1): 105–124. DOI: 10.1007/s10111-017-0453-z.
3. Shahmoradi L, Safadari R and Jimma W (2017) Knowledge Management Implementation and the Tools Utilized in Healthcare for Evidence-Based Decision Making: A Systematic Review. Ethiopian journal of health sciences 27(5): 541–558. DOI: 10.4314/ejhs.v27i5.13.
4. Wu SY, Wang WT and Hsieh YH (2021) "Exploring knowledge sharing behavior in healthcare organizations: an integrated perspective of the empowerment theory and self-determination theory." Kybernetes, in press. DOI: 10.1108/K-01-2021-0028.

**5. The quality of healthcare services and organisational performance are often used interchangeably. The literature review does not sufficiently address the quality (or performance) aspects in a healthcare setting.**

Thanks a lot for the helpful suggestion. You're right. There is partial confusion in the literature, due to the complexity of the phenomenon. We have tried to strengthen this segment of the paper through including literature review which covers various aspects.

Attached please find the references newly included in the revised manuscript for better clarity of our explanations:

1. Busse R, Klazinga N, Panteli D (2020) Improving healthcare quality in Europe: Characteristics, effectiveness and implementation of different strategies. Available from: <https://www.researchgate.net/profile/Guenter_Ollenschlaeger/publication/336739807_Clinical_Practice_Guidelines_as_a_quality_strategy/links/5efdf2fd458515505084bb46/Clinical-Practice-Guidelines-as-a-quality-strategy.pdf>
2. Schroeder P, Parisi LL and Foster R (2019) Healthcare quality improvement: Then and now. *Nursing Management*, *50*(9), 20–25. https://doi.org/10.1097/01.NUMA.0000579004.87116.35
3. Mosadeghrad AM (2013) Healthcare service quality: Towards a broad definition. *International Journal of Health Care Quality Assurance*, *26*(3), 203–219. https://doi.org/10.1108/09526861311311409
4. Endeshaw B (2021) Healthcare service quality-measurement models: a review. *Journal of Health Research*, *35*(2), 106–117. https://doi.org/10.1108/JHR-07-2019-0152

**6. Line 119: In describing the four stages of knowledge creation (SECI model), the word 'internationalisation' needs to be replaced by 'internalisation'.**

Thanks for this correction. We have corrected that in the text..

**7. Since the healthcare services primarily deal with expertise-driven tacit knowledge, 'knowledge sharing' is probably a more appropriate term to use instead of knowledge transfer.**

Thanks for this suggestion. We gladly accepted it and hopefully we have changed it correctly in the document.

We have noticed that there are many discrepancies in the literature regarding the interpretation and use of this term.

For example.

- Tangaraja G, Mohd Rasdi R, Abu Samah B, Ismail M. Knowledge sharing is knowledge transfer: a misconception in the literature. Journal of Knowledge Management. 2016; 20(4):653–70.

**8. The paper refers to ICT as an organisational factor on several occasions (line 15, 65, 456). Do the authors mean the ICT maturity or capability as an organisational factor to influence the knowledge management activities?**

Thanks for this comment. In our text, the authors meant capability as an organizational factor to influence the knowledge management activities.

**9. For the research constructs, the authors mentioned using well-established measuring instruments frequently employed in contemporary research. However, the absence of a few key references indicates methodological weakness.**

You are absolutely right, we apologize for not doing so in the first version of our manuscript. As you may notice in the manuscript, we have added references to this section. In case you think that more corrections are needed in this segment, we are ready to further engage in that direction.

**10. The study involves survey participants from a combination of
institutions across public and private sectors (public: 32 and private:
13). It would have been interesting to see whether an organisation's
characteristics (public vs private) affect its knowledge management
activities.**

Answer to the review: Thank you for your insightful review and relevant suggestion. According to your comment, we focused first on a test model for only public organizations. When constructing our test model for only public organizations, we first checked the standardized factor loadings for all of our four constructs if they are statistically significant (>.50). One item intended to measure knowledge creation and two items intended to measure quality of healthcare services and information communication technologies did not meet the criteria recommended in the literature and were therefore omitted from the “final” version of the public test model. Our “final” public test model consists of 15 items utilized to evaluate the existing state of four measured constructs. Additionally, we evaluated the model fit to data. Fit indices are as follows: CFI = 0.96 (in original model proposed in the manuscript 0.97); chi-square 120.957 (in original model proposed in the manuscript 104.690); RMSEA = .07 (in original model proposed in the manuscript.07); and df = 77 (in original model proposed in the manuscript 67). Fit indices are therefore satisfactory.

We then ran a series of hierarchical regression analysis utilizing centered variables to test our conceptual public test model. The hypotheses are explained in our original research in Figure 1.

The statistical comparison of “Model 1”, related to hypothesis is presented below:

|  | Original model | Public |
| --- | --- | --- |
| R^2^ | 0.369 | .217 |
| ß | .604 | .356 |
| sig | .000 | .002 |

The comparison of “Model 2”, related to hypothesis 2 is presented below:

|  | Original model | Public |
| --- | --- | --- |
| R^2^ | 0.404 | .275 |
| ß | .660 | .475 |
| sig | .000 | .000 |

The comparison of “Model 3”, related to hypothesis 3 is presented below:

|  | Original model | Public |
| --- | --- | --- |
| R^2^ | 0.404 | .227 |
| ß | .210 | .119 |
| sig | .008 | .315 – not significant |
| ΔR^2^ | .035 | .010 – less added value of interaction |

The comparison of “Model 4”, related to hypothesis 4 is presented below:

|  | Original model | Public |
| --- | --- | --- |
| R^2^ | 0.483 | .313 |
| ß | .240 | .230 |
| sig | .001 | .040 – lower value |
| ΔR^2^ | .047 | .038 – less added value of interaction |

Every model that was tested only in public organizations shows a lower R^2^ value, indicating that the proportion of the variance for our dependent variable that is explained by our independent variable is lower. In addition, in model 3 and 4 there is a smaller change in R^2^, indicating that there is less added value by the interaction effect. Moreover, in model 3 the interaction is even not significant.

When constructing our model for only private organizations, we again first checked the standardized factor loadings for all of our four constructs if they are statistically significant (>.50). Four items intended to measure knowledge transfer, two items intended to measure quality of healthcare services and two items intended to measure information communication technologies did not meet the criteria in the literature and were therefore omitted from the “final” version of the private test model. One item was conditionally included in the measurement of information communication technologies as it would not be possible to include our interaction variable with only one question, even if the standardized factor loading was below the recommended value. Our “final” private model consists of 12 items utilized to evaluate the existing state of four measured constructs. The model fit to data is as follows: CFI = 0.80 (original model 0.97); chi-square 86.745 (original model 104.690); RMSEA = .162 (original model .07); and df = 43 (original model 67). As CFI and RMSEA are below the recommended values in the literature, we concluded that it does not make sense to run any further tests on this specific model.

Therefore, we conclude that there are indeed important differences between public and private organizations that affect knowledge management activities. However, we believe that the original model, combining public and private organizations better suits our research study and proposed constructs and adds more scientific value to the research community.

***Answer to the reviewer:*** *We would like to thank you for your precise and insightful revision. We have rewritten part of the text according to your valuable propositions. You helped us a lot by opening many important topics that needed to be addressed in our research. Your instructions were integral to partially restructure the whole paper, with our intention to fully follow your comments. Sincerely thank you for your effort and clear instructions.*
